# Systemic tryptophan homeostasis

**DOI:** 10.3389/fmolb.2022.897929

**Published:** 2022-09-14

**Authors:** Simon Klaessens, Vincent Stroobant, Etienne De Plaen, Benoit J. Van den Eynde

**Affiliations:** ^1^ Ludwig Institute for Cancer Research, Brussels, Belgium; ^2^ de Duve Institute, UCLouvain, Brussels, Belgium; ^3^ Walloon Excellence in Life Sciences and Biotechnology, Wavre, Belgium; ^4^ Nuffield Department of Clinical Medicine, Ludwig Institute for Cancer Research, University of Oxford, Oxford, United Kingdom

**Keywords:** tryptophan, TDO (tryptophan 2,3-dioxygenase), IDO1 (indoleamine 2,3-dioxygenase 1), pellagra, hartnup disease, tumor, SLC6A19, SLC16A10

## Abstract

Tryptophan is an essential amino acid, which is not only a building block for protein synthesis, but also a precursor for the biosynthesis of co-enzymes and neuromodulators, such as NAD/NADP(H), kynurenic acid, melatonin and serotonin. It also plays a role in immune homeostasis, as local tryptophan catabolism impairs T-lymphocyte mediated immunity. Therefore, tryptophan plasmatic concentration needs to be stable, in spite of large variations in dietary supply. Here, we review the main checkpoints accounting for tryptophan homeostasis, including absorption, transport, metabolism and elimination, and we discuss the physiopathology of disorders associated with their dysfunction. Tryptophan is catabolized along the kynurenine pathway through the action of two enzymes that mediate the first and rate-limiting step of the pathway: indoleamine 2,3-dioxygenase 1 (IDO1) and tryptophan 2,3-dioxygenase (TDO). While IDO1 expression is restricted to peripheral sites of immune modulation, TDO is massively expressed in the liver and accounts for 90% of tryptophan catabolism. Recent data indicated that the stability of the TDO protein is regulated by tryptophan and that this regulation allows a tight control of tryptophanemia. TDO is stabilized when tryptophan is abundant in the plasma, resulting in rapid degradation of dietary tryptophan. In contrast, when tryptophan is scarce, TDO is degraded by the proteasome to avoid excessive tryptophan catabolism. This is triggered by the unmasking of a degron in a non-catalytic tryptophan-binding site, resulting in TDO ubiquitination by E3 ligase SKP1-CUL1-F-box. Deficiency in TDO or in the hepatic aromatic transporter SLC16A10 leads to severe hypertryptophanemia, which can disturb immune and neurological homeostasis.

## 1 Introduction

**FIGURE 1:**
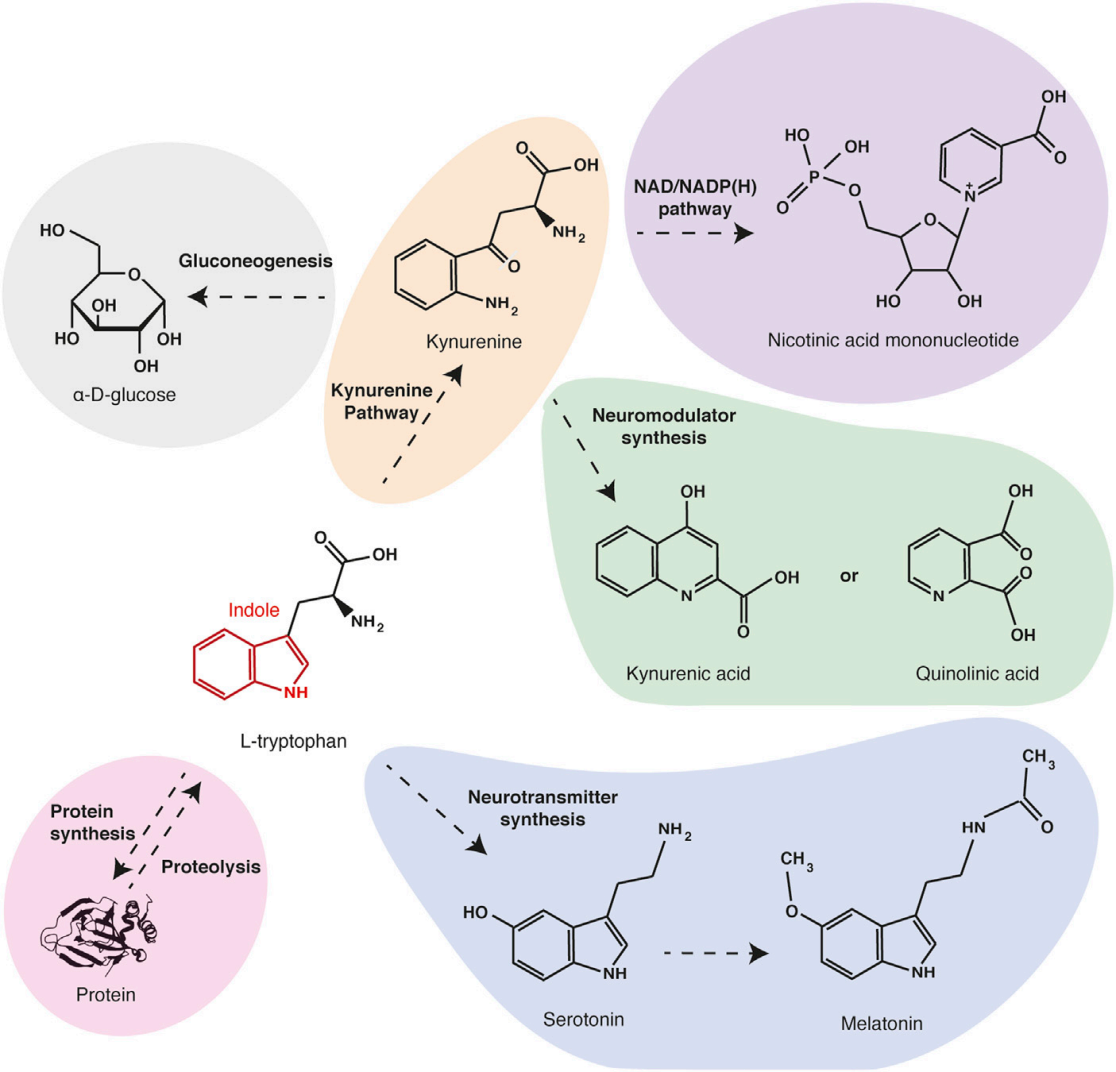
Main tryptophan metabolites and functions. Tryptophan is one of the twenty proteinogenic amino acids. Besides being a protein building block, tryptophan can be oxidized in kynurenine (orange), the precursor of electron transporters NAD/NADPH (purple) and of neuromodulators quinolinic acid and kynurenic acid (green). Through the same pathway, tryptophan can also be metabolized in alanine and serve as a substrate for gluconeogenesis (grey). Alternatively, tryptophan can be metabolized in neurotransmitters serotonin and melatonin (blue).

L-tryptophan is a neutral and aromatic molecule, which is one of the nine essential proteinogenic amino acids in mammals. Tryptophan is the only one to have an indole group, a bicyclic structure consisting of a benzene ring fused to a pyrrole (Palego et al., 2016). This chemical structure gives a high hydrophobicity and stability to the tryptophan and makes it the proteinogenic amino acid with the longest carbon skeleton. Due to these chemical particularities, tryptophan and its metabolites also serve as biochemical precursors for many compounds (Figure 1). Quinolinic acid, the main tryptophan metabolite along the kynurenine pathway, is the precursor of nicotinamide and NAD/NADP(H). Thus, an unbalanced diet that is poor in tryptophan can induce nicotinamide deficiency causing a metabolic disorder called pellagra. Through the kynurenine pathway, tryptophan is also metabolized in alanine, which can serve as a substrate for gluconeogenesis. Tryptophan is also the precursor of neuroactive compounds serotonin and melatonin, which are involved in the regulation of behavioral controls and circadian rhythms (Lewy et al., 1992; Mosienko et al., 2012), and of quinolinic acid and kynurenic acid, which are agonist and antagonist of glutamate receptors, respectively (Stone and Perkins, 1981; Perkins and Stone, 1982; Schwarcz et al., 2012). Thus alterations of tryptophan concentration and metabolism are involved in psychological disorders.

Tryptophan biosynthesis in only ensured by plants, fungi and microorganisms (Parthasarathy et al., 2018). As other aromatic amino acids, tryptophan is synthesized from erythrose 4-phosphate, a pentose phosphate pathway intermediate, or from phosphoenolpyruvate, a glycolytic intermediate, which are both cyclized in chorismate. Tryptophan synthesis is energetically expensive and requires the action of many enzymes and biochemical steps. This particularity probably explains why among the twenty proteogenic amino acids, tryptophan is the least abundant residue in most proteins. Indeed, it accounts only for 1% to 2% of the total amino acid content in animal proteins (Kaluzna-Czaplinska et al., 2019). Consequently, this amino acid is chemically precious and its bioavailability is important in the struggle for life. Tryptophan levels play a key role in anti-microbial immune responses. Indeed, local tryptophan depletion induced by immune cells during inflammation was found to inhibit the proliferation of pathogens (Pfefferkorn et al., 1986b; Schmidt et al., 2009).

Moreover, tryptophan catabolism through the kynurenine pathway plays a leading role in immune tolerance, preventing immunopathology due to uncontrolled or overreacting immune responses. On the one hand, local tryptophan depletion has been shown to inhibit T cell proliferation (Munn et al., 2005). This could be due to GCN2 (General control amino acid non-derepressible 2) activation triggered by the accumulation of unfilled tRNA (Munn et al., 2005) or mTOR (Mammalian target of rapamycin) inactivation in response to tryptophan starvation (Metz et al., 2012). On the other hand, accumulation of tryptophan catabolites can induce T cell apoptosis or promote their differentiation in Treg (Regulatory T cells) (Terness et al., 2002; Fallarino et al., 2006). Recent discoveries suggest that kynurenine and derivatives act as immunosuppressors through the binding and the activation of the aryl hydrocarbon receptor (AHR) (Opitz et al., 2011). Many tumors hijack the expression of tryptophan-degrading enzymes to prevent their immune rejection (van Baren and Van den Eynde, 2015a). The role of tryptophan catabolism in immunotolerance and cancer immunity will be detailed later.

The essential functions of tryptophan in metabolism, neurology and immunity explain why its systemic concentration needs to be constant. In humans, tryptophan plasmatic concentration, or tryptophanemia, is strictly maintained at 60 ± 15 µM (Armstrong and Stave, 1973; Stegink et al., 1991; Lepage et al., 1997; Geisler et al., 2015). Tryptophan homeostasis involves four complementary steps to regulate its systemic concentration: 1) prandial intake, 2) intestinal absorption and renal reabsorption, 3) protein synthesis and degradation and 4) amino-acid catabolism.

## 2 Dietary tryptophan consumption

In healthy human adults, the amount of dietary proteins ranges from 1,000 to 1,400 mg/kg/day, a quantity significantly higher than the protein Recommended Dietary Allowance (RDA) of 660 mg/kg/day (Trumbo et al., 2002). Given the low tryptophan content in proteins, we eat on average approximately 12 mg/kg/day of tryptophan, while the RDA is 5 mg/kg/day. These values are given for American adult men and women and can vary depending on age and countries. Usually, symptoms associated with amino‐acid deficiency are due to severe protein malnutrition, which does not allow to determine the function of each amino acid separately. However, tryptophan deficiency has been clearly associated with a specific disorder, the pellagra (Bender, 1983; Pitche, 2005). This disease is caused by a severe deficiency in tryptophan and the two forms of vitamin B3 (nicotinic acid and nicotinamide), leading to decreased synthesis of NAD/NADP(H). Considered for a long time as an infectious disease, pellagra reached epidemic proportions in the 19^th^ and 20^th^ centuries and caused the death of hundreds of thousand people around the world. Common in poor populations with an exclusive maize diet, this disease is due to the low content of tryptophan in maize proteins and the failure of those who introduced maize from Central and South America in Southern Europe to follow the liming process that results in the release of the abundant vitamin B3 from its polysaccharide-bound form, niacytin, which cannot be hydrolyzed by mammalian digestive enzymes (Bender, 1983).

Clinically, pellagra is characterized by three symptoms: dermatitis, diarrhea and dementia (Bender, 1983; Pitche, 2005). The disease begins with typical erythematous lesions on photo-exposed skin areas, bilaterally distributed on the face, neck and back of the hands. This fragility of the skin gives its name to the disorder from the Italian “pelle = skin” and “agra = rough”. Symptoms are getting worse with the development of chronic diarrheas leading to dehydration, nausea, anorexia and cachexia. In the last stages of the disease, subjects suffer from neuropsychological disorders characterized by depression, hallucination, memory loss and psychosis, which lead to dementia, and in most severe cases, to death. The biochemical causes underlying pellagra symptoms remain poorly understood. On the one hand, Vasantha and colleagues showed that the concentration of histidine and its metabolite urocanic acid decreased in the skin of pellagra patients (Vasantha, 1970). As urocanic acid located in the stratum corneum of the epidermis acts as a photoprotectant against UVB-induced-DNA damage, its disappearance would explain the dermatitis. However, the underlying link with vitamin B3 or tryptophan has not been identified. On the other hand, vitamin B3 and tryptophan deficiency could reduce the synthesis of NAD/NADP(H), two essentials cofactors involved in cellular energy transfer reactions. As a consequence, their decrease could impact the metabolic activity of high-energy demanding tissues, such as the brain, or of frequently regenerating tissues, such as the intestine mucosa and the skin (Harber and Bickers, 1989).

In contrast, tryptophan-rich diets or food supplements have a moderate impact on health. Fernstrom and colleagues showed in human subjects that oral administration of juice containing 40 g of α-lactalbumin, a protein rich in tryptophan, increased 3-fold its plasmatic concentration over fasting within 90 min, which declined rapidly by 240 min (Fernstrom et al., 2013). Thus, tryptophan prandial intake does not impact its systemic concentration on the long term. As tryptophan is the precursor of melatonin and serotonin, several studies have investigated the consequences of oral tryptophan administration on sleep and behavior. Tryptophan food complements have been shown to reduce the sleep latency, improve moderately the cognition and modulate mood in volunteers (Silber and Schmitt, 2010). These observations correlate with the serotonin increase observed in the plasma and brain of rat fed with tryptophan complements (Mateos et al., 2009).

## 3 Intestinal and renal absorption of tryptophan

### 3.1 Amino-acid transport across polarized cells

**FIGURE 2:**
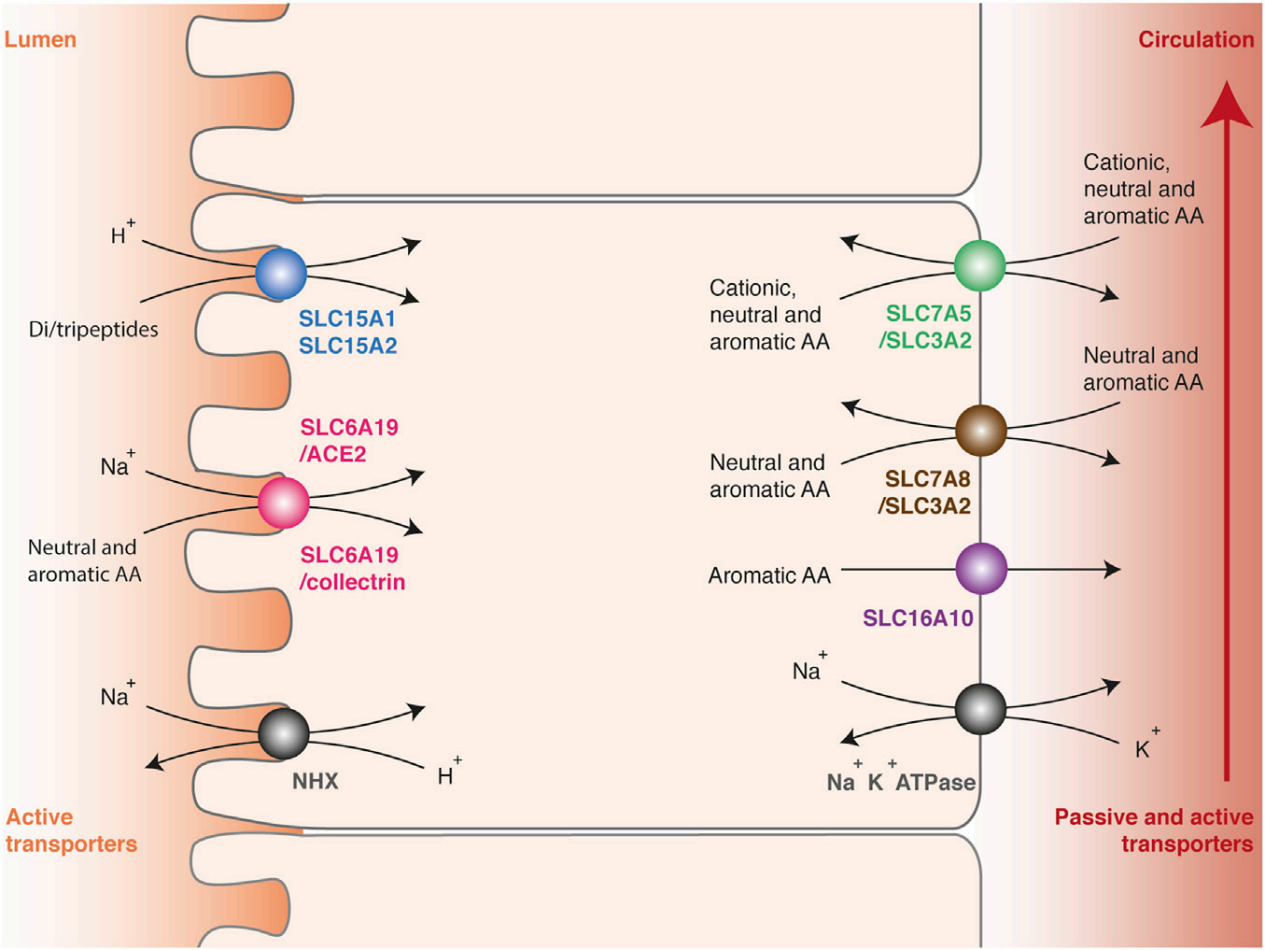
Intestinal absorption and renal reabsorption of tryptophan. Transport of tryptophan involves two successive steps across polarized cells. First, tryptophan is transported against the concentration gradient from the lumen to the cytosol of epithelial cells by apical transporters using the electrochemical potential gradient of sodium ion or proton (left side). Tryptophan is captured in peptidic or free form, by di-/tripeptides transporters SLC15A1 and SLC15A2 (blue) or by neutral amino acid transporters SLC6A19/ACE2 and SLC6A19/collectrin (pink), respectively. Secondly, tryptophan is released from the cytosol to the blood through basolateral transporters (right side), namely the multiple amino acid exchangers SLC7A5/SLC3A2 (green) and SLC7A8/SLC3A2 (brown), or the passive aromatic transporter SLC16A10 (purple). The electrochemical gradient of protons (pH < 6.5) is maintained by the apical Na^+^/H^+^ exchanger NHX (grey, left side). The electrochemical gradient of sodium is maintained by the basolateral Na^+^/K^+^ ATPase (grey, right side).

Once ingested, dietary proteins are initially degraded to oligopeptides by gastric pepsins and pancreatic proteases in the small intestine (Stipanuk and Caudill, 2013). The breakdown products are then hydrolyzed a second time to di- or tripeptides or free amino acids by peptidases anchored in the microvillus membrane. As for glucose, their transport from the intestinal lumen to the portal vein involves two successive steps across polarized cells (Kandasamy et al., 2018). Amino acids are initially captured at the apical membrane against the concentration gradient by active transporters to ensure a complete absorption of nutrients (Figure 2). Their accumulation involves secondary active transporters, which convey their substrates by using the electrochemical potential gradient of sodium ion or proton. Low sodium content in the cytosol is maintained by the basolateral Na^+^/K^+^ ATPase, while the acidic pH (<6.5) at the brush border membrane surface is maintained by the multiple apical Na^+^/H^+^ exchanger NHX (Sodium/hydrogen exchanger). Once concentrated in enterocytes, amino acids are released in the portal vein by facilitated diffusion or exported by secondary active transporters at the basolateral membrane. Intestinal absorption of amino acids works in a similar way as the reabsorption in kidneys, and many transmembrane transporters are common between enterocytes and epithelial cells of the renal tubules.

Amino-acid transporters have been regrouped in a solute carrier family (SLC) and classified according to the Hugo Nomenclature Committee rules relative to sequence identities (Hediger et al., 2004). Amino acids and peptides are selectively transported depending on their physicochemical properties and their carbon skeleton. In enterocytes and epithelial cells of renal tubules, three apical transporters able to capture tryptophan have been described: SLC15A1 (previously called PEPT1), SLC15A2 (PEPT2) and SLC6A19 (B^0^AT1) (Figure 2) (Broer and Broer, 2017; Kandasamy et al., 2018). These proteins play a key role in tryptophan absorption, but also ensure the transport of many other amino acids. The monomeric transporters SLC15A1 in enterocytes and SLC15A2 in epithelial cells of renal tubules mediate the H^+^-dependent transport of di- or tripeptides. These transporters are not specific for one amino acid, probably because they recognize the universal backbone of peptides rather than the lateral chain of amino acids (Martinez Molledo et al., 2018). SLC6A19 forms a heterodimeric complex with ACE2 (Angiotensin-converting enzyme 2) in enterocytes (Camargo et al., 2009; Yan et al., 2020) or collectrin in epithelial cells of renal tubules (Danilczyk et al., 2006). SLC6A19/ACE2 and SLC6A19/collectrin transporters facilitates the Na^+^-dependent transport of a broad range of neutral and aromatic amino acids. Active reabsorption of these amino acids is particularly efficient, since only 0.2% of the tryptophan ingested in healthy subjects is eliminated through the urinary tract (Terakata et al., 2013).

Although several transporters mediate tryptophan absorption and reabsorption, SLC6A19/ACE2 and SLC6A19/collectrin play a preponderant role. Indeed, inactivating mutations of SLC6A19 cause the Hartnup disorder, which is mainly characterized by a tryptophan deficiency (Bender, 1983; Kleta et al., 2004). This autosomal recessive disease manifests by a neutral hyperaminoaciduria and the accumulation of tryptophan catabolites produced by bacteria, indole and tryptamine, in the intestinal tract. In most patients, the disorder remains asymptomatic due to a protein-rich diet and the alternative transport of tryptophan included in oligopeptides. With an inadequate or poor diet supply, subjects suffer from tryptophan deficiency characterized by photosensitive skin affections, ataxia and neurological disorders, which are reminiscent of pellagra symptoms. In consistence with SLC6A19 deficiency, ACE2 KO mice present intestinal Hartnup symptoms (Camargo et al., 2009), while collectrin KO mice present renal Hartnup symptoms (Danilczyk et al., 2006).

Once concentrated into the cytosol, tryptophan is exported in the blood circulation by three basolateral transporters common in enterocytes and epithelial cells of renal tubules: SLC7A5/SLC3A2 (LAT1/CD98hc: Large neutral amino acid transporter 1/CD98 heavy chain), SLC7A8/SLC3A2 (LAT2/CD98hc) and SLC16A10 (TAT1: T-amino acid transporter 1) (Figure 2) (Stipanuk and Caudill, 2013; Napolitano et al., 2015; Broer and Broer, 2017; Kandasamy et al., 2018). SLC7 or LAT proteins are usually referred to as transporters, but only constitute the functional subunit of heterodimeric complexes. The functional transporters are constituted of a structural protein (heavy chain) from the SLC3 family and an active subunit (light chain) from the SLC7 family, linked together by a disulfide bond. SLC7A5/SLC3A2 ensures the transport of aromatic, neutral or cationic amino acids, and SLC7A8/SLC3A2 the transport of aromatic and neutral amino acids except proline. Both are amino-acid exchangers or antiporters: for one amino acid transferred into the cytosol, one is expulsed outside. Therefore, they equilibrate the amino-acid distribution across the membrane, in contrast with symports, which ensure net amino acids efflux. SLC16A10 is a monomeric symport mediating the passive diffusion of all aromatic amino acids from the cytosol to the blood circulation. Interestingly, SLC16A10 KO mice do not suffer from tryptophan deficiency or symptoms common with pellagra, even when fed with a low protein diet, indicating that this transporter is not essential for tryptophan absorption and reabsorption (Mariotta et al., 2012).

### 3.2 Nutrient-sensing G-protein coupled receptors

In intestinal absorption, the mechanisms have evolved to avoid loss of nutrients by adapting their transport to diet composition. As a result, dietary protein intake can increase rapidly the systemic concentration of amino acids. To anticipate this increase, amino-acid inflow is sensed along the digestive tract by amino-acid sensitive G-protein coupled membrane receptors (GPCR), which are expressed in the taste buds and gastrointestinal tract (Conigrave and Hampson, 2010; Wauson et al., 2013; Ahmad and Dalziel, 2020). These receptors are also expressed in β cells of pancreatic Langerhans islets, where they detect the increase of blood amino-acid levels in the postprandial period (Oya et al., 2011; Wauson et al., 2013). They trigger the production of hormones that control amino-acid capture, protein synthesis and satiety. These receptors are histo-specific and sensitive to extracellular concentration of amino acids, in contrast with the ubiquitous mTOR and GCN2 signaling pathways, which sense intracellular levels.

Amino-acid sensitive GPCRs comprise a Venus-flytrap ligand binding segment, which can accommodate many agonists (Conigrave and Hampson, 2010; Wauson et al., 2013; Ahmad and Dalziel, 2020). Three GPCRs capable of detecting a wide variety of amino acids have been identified: GPRC6A (G Protein-coupled Receptor family C 6A), TAS1R1/TAS1R3 heterodimeric complex (Taste Receptor type1 and 3) and CasR (Calcium-sensing receptor). Their signal transduction has not yet been described, but causes the release of incretins and peptide YY (pyy) by gastrointestinal cells, and of insulin by pancreatic islets. In the postprandial period, insulin, whose secretion is enhanced by incretins, promotes mTORC1 activation (see below), which stimulates the storage of amino acids by increasing protein synthesis (Laplante and Sabatini, 2012). Furthermore, pyy increases satiety by activating GPCR Y2 in hypothalamic cells, which control appetite and energy balance (Loh et al., 2015).

Interestingly, Lin and colleagues recently identified GPCR 142 (GPR142) as a specific sensor of L-tryptophan (Lin et al., 2016). Treatment of human GPR142-transfected cells with tryptophan increased inositol 1-phosphate concentration (IP-1) in a-dose dependent manner and had no effect on untransfected control cells. Furthermore, the gavage of fasting mice with tryptophan induced secretion of insulin by the β cells of Langerhans islets and of gastric inhibitory polypeptide incretin (GIP) by duodenal K cells in wild-type mice, but not in GPR142-KO mice. The hormones released by GPR142 activation had a strong impact on glycemia when tryptophan was co-administrated with glucose. The protein synthesis and systemic concentration of amino acids were not evaluated, but are usually influenced by these hormones through their effect on mTORC1. GPR142-KO mice revealed no other obvious signs, probably due to the redundancy of alternative signaling pathways sensitive to amino acids.

## 4 Amino-acid storage and protein turnover

To increase glycemia in fasting periods, the liver can generate glucose by synthesizing it through gluconeogenesis or by hydrolyzing glycogen. If necessary, alternative sources of energy, such as fatty acids or ketone bodies, can partially replace glucose. These mechanisms are not applicable to amino acids, because some of them are essential. However, large quantities of amino acids are stored in proteins, which can be degraded to maintain homeostasis. Muscles, which represent approximately 40% of the human body mass, constitute the largest reservoir.

Muscle proteins are subject to a rapid turnover depending on diet supply. After consumption of a protein-containing meal, amino-acid afflux promotes muscular growth. Fulks and colleagues initially showed that addition of a complete mixture of amino acids to *ex vivo* cultures of diaphragm increased the muscular protein mass, by stimulating protein synthesis and inhibiting catabolism (Fulks et al., 1975). Interestingly, the same effect was obtained by the provision of branched amino acids alone (leucine, isoleucine and valine), while other amino acids together had a moderate impact. Subsequent studies covered by Jefferson confirmed, by incorporation of radioactive phenylalanine, that a decrease in muscular protein synthesis induced by fasting in rat, could be rescued by the administration of leucine alone (Anthony et al., 2000). Recovery of protein synthesis was associated with the phosphorylation of 4E-BP1 and S6K, both involved in translation initiation, which highlights the role of mTOR in protein turnover. When amino acids are abundant, the mTOR signaling pathway remains active and promotes translation, thereby stimulating cell proliferation, growth and survival (Laplante and Sabatini, 2012). In contrast, when amino acids become scarce, the mTOR signaling pathway is inactivated, which decreases protein synthesis and promotes autophagy. Like that of all other amino acids, tryptophan homeostasis is influenced by protein turnover. However, tryptophan is not the actor. Indeed, leucine is the primary activator of mTOR, maybe because leucine is both an essential amino acid and the most common proteinogenic amino acid (Ananieva et al., 2016).

### 4.1 Postprandial protein synthesis

**FIGURE 3:**
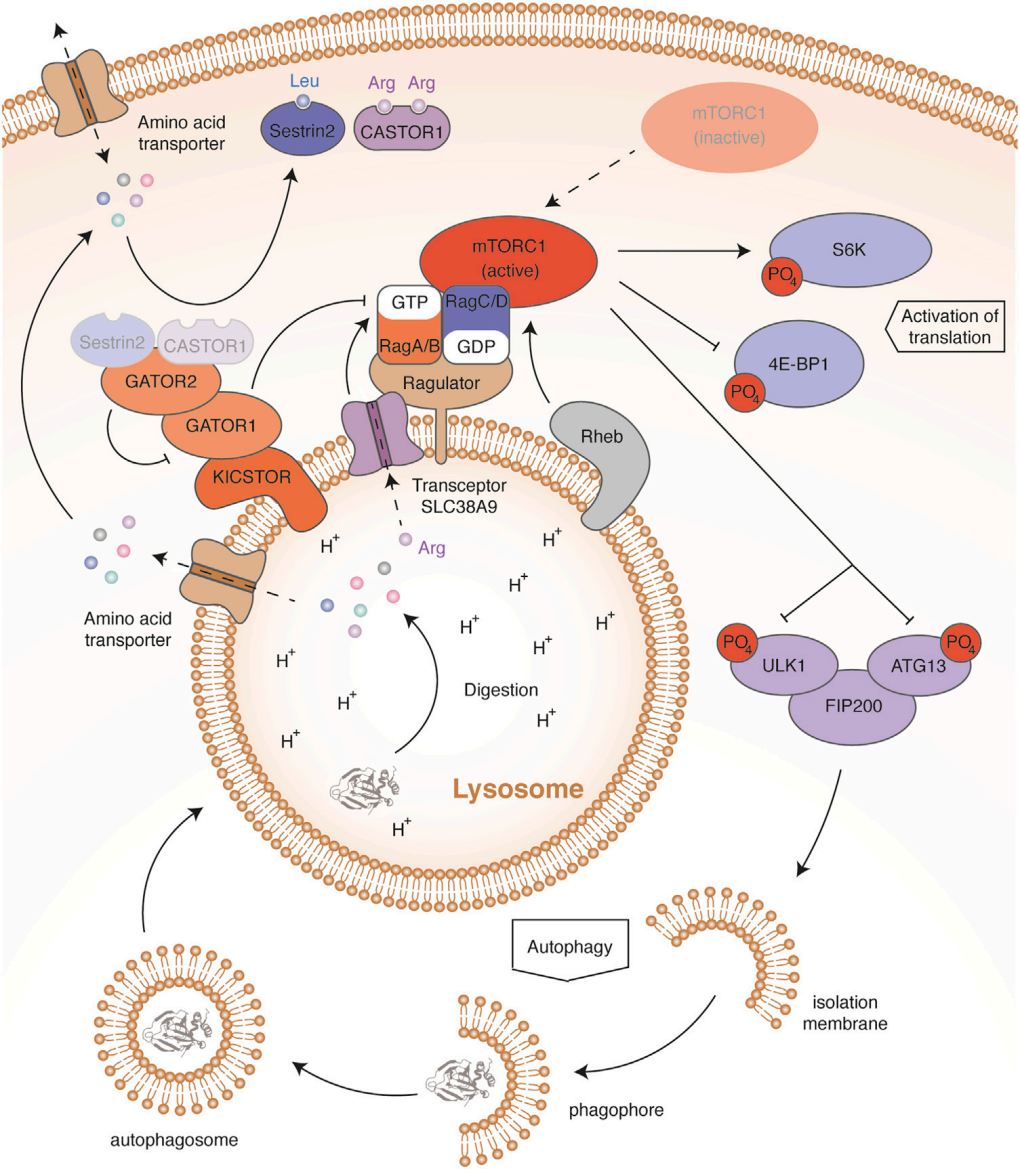
mTORC1 in amino acid regulation. In high amino acid conditions, Sestrin2 and CASTOR1 bind leucine and arginine, respectively. This induces their dissociation from GATOR2. Once released, GATOR2 inhibits by an unknown mechanism the activity of GATOR1, thereby preventing activation of the GTPase activity of RagA and RagB. In addition, high arginine levels sensed through the lysosomal transceptor SLC38A9 induce the release of GDP from RagA and RagB. High amino acid levels therefore maintain the binding of RagA and RagB to GTP. This allows Rags to recruit the mTORC1 complex to the lysosomal membrane, thereby allowing activation of the mTOR kinase by Rheb. The mTOR activation contributes to amino acid storage and consumption by activating protein synthesis through the phosphorylation of S6K and 4E-BP1. mTOR activation also blocks autophagy by phosphorylating the ULK1-ATG13-FIP200 complex, stopping the recycling and release of amino acids. Conversely, in low amino acid conditions, the mTORC1 complex remains free and inactive in the cytosol. Absence of S6K and 4E-BP1 phosphorylation impairs protein synthesis, contributing to amino acid saving. Dephosphorylation of the ULK1-ATG13-FIP200 complex initiates autophagy and lysosomal protein degradation, releasing free amino acids.

The mTOR protein is a serine-threonine kinase that forms the core of a multi-protein complex called mTORC1 (Mammalian target of rapamycin complex 1) (Figure 3). Sabatini’s group showed that an increase in leucin mediates mTORC1 activation through cytosolic Rag GTPases (Ras-related GTP-binding protein) (Sancak et al., 2008; Sancak et al., 2010). These proteins are recruited from the cytosol by the Ragulator complex anchored in the lysosomal membrane. The four Rag proteins assemble as heterodimers, consisting of RagA or RagB with RagC or RagD. The two proteins composing the complex bind a nucleotide with a different phosphorylation state: when RagA or RagB binds GDP, then RagC or RagD binds GTP and vice versa. An increase in the leucine concentration promotes the binding of RagA or RagB to GTP. This change allows Rags to recruit RAPTOR (Regulatory-associated protein of mTOR), a subunit of the mTORC1 complex. The Rag proteins that interact with the lysosomal protein membrane Ragulator thus induce a relocation of the mTORC1 complex close to the small GTPase Rheb (Rag homologue enriched in brain). Rheb then activates the mTOR kinase through an unknown mechanism. Rheb activity requires its binding to GTP, which is conditioned by growth signaling, through the PI3K-AKT pathway (Phosphoinositide 3-kinase/RAC-alpha serine/threonine-protein kinase). This ensures that mTOR activation occurs only if all requirements for cell growth are fulfilled (Condon and Sabatini, 2019). Once activated, the mTOR kinase stimulates translation by activating the S6K ribosomal protein and by inhibiting the factor 4E-BP1 (Eukaryotic translation initiation factor 4E-binding protein 1) through phosphorylation (Sancak et al., 2008).

The amino-acid sensors mediating mTOR activation are not fully described but regulate the nucleotide-bound state of Rags (Figure 3) (Bar-Peled et al., 2013). GATOR1 (GAP activity toward the Rag GTPases 1) stimulates the GTPase activity of RagA and RagB, resulting in mTORC1 inhibition. To interact with Rags, GATOR1 is anchored in the lysosomal membrane by the multiprotein scaffold KICSTOR (Peng et al., 2017; Wolfson et al., 2017). Deficiency of KICSTOR components mislocates GATOR1, which leads to an amino-acid insensitive mTOR pathway. In addition, GATOR1 interacts with GATOR2 (GAP activity toward the Rag GTPases 2), a multiprotein complex inhibiting the GTPase-accelerating activity of GATOR1. Two amino-acid sensors have been described, Sestrin2 and CASTOR1 (Cytosolic arginine sensor of mTORC1 subunit 1), sensitive to leucin and arginine concentrations, respectively (Chantranupong et al., 2016; Kimball et al., 2016; Wolfson et al., 2016). Upon amino-acid starvation, Sestrin2 and CASTOR1 bind and inhibit GATOR2, leading to mTORC1 inhibition. When the amino acids are abundant, Sestrin2 and CASTOR1 directly bind leucin and arginine, respectively, and dissociate from GATOR2, resulting in mTORC1 activation. In addition, arginine abundance can modulate the activity of mTOR through the lysosomal amino-acid transporter SLC38A9 (Jung et al., 2015; Rebsamen et al., 2015; Wang et al., 2015). Such nutrient transporters, which communicate the abundance of their substrate, have been termed transceptors (Broer and Broer, 2017). In high arginine levels, transceptor SLC38A9 interacts with the Ragulator complex and triggers the GDP release from RagA/B, promoting the activation of mTORC1. Leucine and arginine seem to play a preponderant role in the mTOR signaling pathway. Besides the intracellular effect of these amino acids, post-prandial insulin secretion promotes mTOR activation through the PI3K/AKT signaling pathway. Indeed, active AKT ensures the phosphorylation of TSC complex, which dissociates from Rheb, thereby activating it (Sancak et al., 2008; Condon and Sabatini, 2019). In this way, the activation of GPCRs sensitive to dietary amino acids in epithelial cells of the digestive system and in β cells of Langerhans islets, such as TAS1R1/TAS1R3 or GPR142, also contribute to amino-acid storage. Through insulin, all amino acids can therefore influence muscle protein synthesis.

### 4.2 Amino-acid release by protein autophagy

During fasting, protein autophagy generates amino acids, in order to sustain their systemic concentration. Hosokawa and colleagues showed in human cell lines that mTORC1 interacts with a pro-autophagic complex composed of ULK1 kinase (Unc-51 like activating kinase 1), Atg13 (Autophagy-related protein 13) and FIP200 (Family kinase-interacting protein of 200 kDa) (Hosokawa et al., 2009). In complete medium, mTOR kinase inhibits autophagy by phosphorylating ULK1 and ATG13 (Figure 3). By contrast, in amino-acid free medium, mTOR inactivation leads to a progressive dephosphorylation of ULK1 and ATG13, which initiates autophagosome formation. Autophagy dysregulation induced by the deficiency of several ATG family members (Autophagy-related proteins) was shown to decrease the plasma levels of essential amino acids in fasting newborn mice (Kuma et al., 2004; Komatsu et al., 2005; Sou et al., 2008).

In the post-prandial period, the increase of circulating amino acids and the release of insulin activate the mTOR signaling pathway, which stimulates protein synthesis. The storage of amino acids in proteins decreases their systemic concentration. Under fasting, the shortage of amino acids inactivates mTOR, inducing autophagy and decreasing protein synthesis. This recycling of amino acids allows to maintain their systemic concentration. The contribution of protein turnover in amino-acid homeostasis relative to other mechanisms remains to be defined.

## 5 Tryptophan catabolism

In contrast to other mechanisms involved in amino-acid homeostasis, the enzymes involved in their metabolism or catabolism are selective and can regulate their concentration independently of one another. In humans, approximately 95% of overall tryptophan is degraded through the kynurenine metabolic pathway, whose first reaction is catalyzed by TDO (Tryptophan 2,3-dioxygenase) or IDO1 (Indoleamine 2,3-dioxygenase 1) (Badawy, 2017). Tryptophan can also be metabolized in 5-hydroxy-L-tryptophan, a serotonin precursor, by TPH1 and TPH2 (Tryptophan 5-hydroxylase 1 and 2) (Hamon et al., 1981). As their activities have a minor impact on tryptophan homeostasis, they will not be reviewed here.

### 5.1 The kynurenine pathway

**FIGURE 4:**
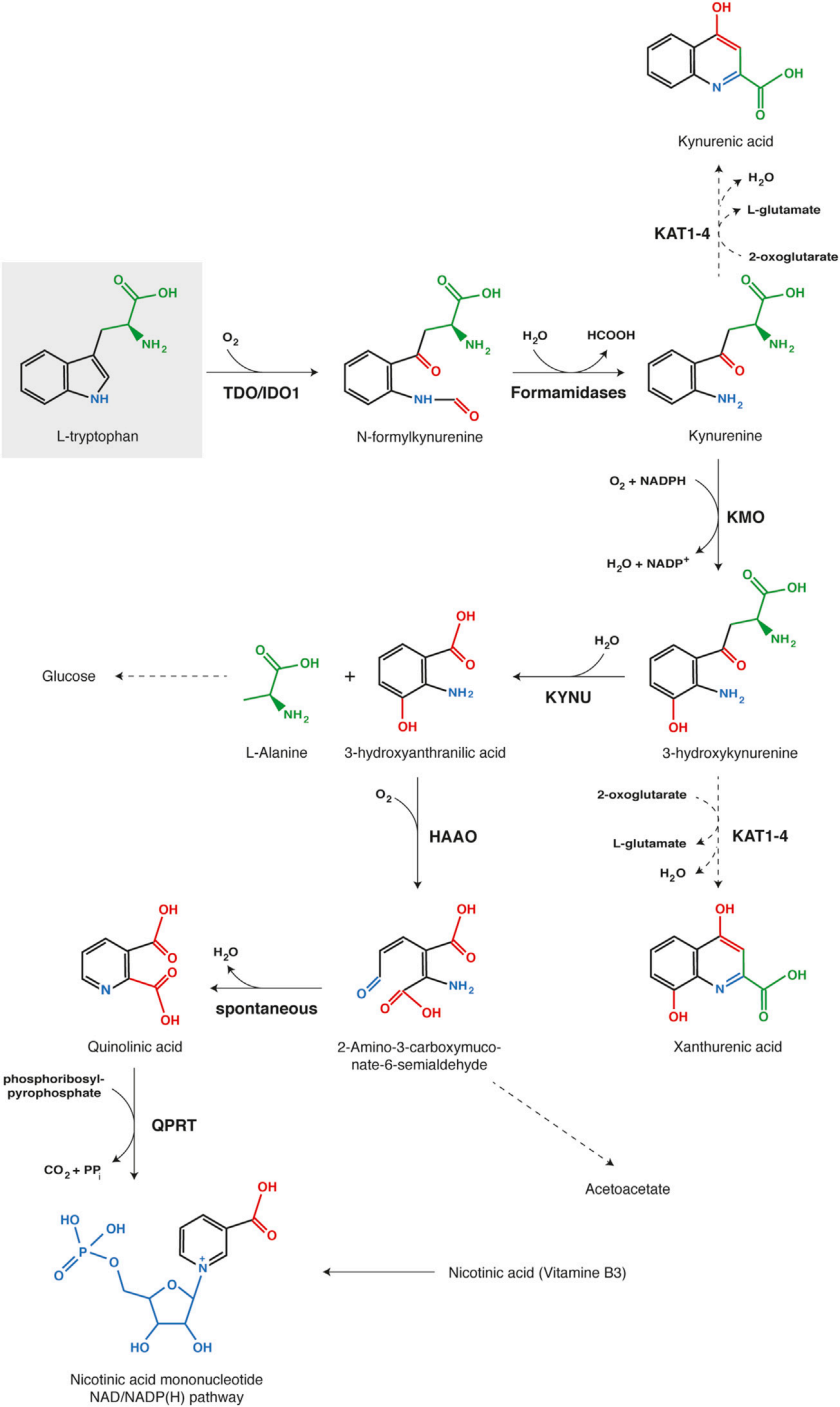
Kynurenine pathway of tryptophan catabolism. Each chemical function modified along the process is indicated with a different color (red, blue or green). The first reaction, tryptophan oxidation by IDO1 or TDO, is the limiting step of the metabolic pathway. Abbreviations: TDO, Tryptophan 2,3-dioxygenase; IDO1, Indoleamine 2,3-dioxygenase 1; KMO, kynurenine 3-monooxygenase; KYNU, kynureninase; HAAO, 3-hydroxy-anthranilate 3,4-dioxygenase; QPRT, Quinolinate phosphoribosyl transferase; KAT1-4, Kynurenine aminotransferase 1-4.

Through the kynurenine pathway, tryptophan can be completely degraded in alanine and acetoacetate or metabolized in biologically active compounds such as NAD/NADP(H) (Figure 4). The metabolic pathway starts with the oxidative breakdown of the L-tryptophan indole group by TDO or IDO1 to produce N-formylkynurenine, which is rapidly hydrolyzed in kynurenine by formamidases (Voet et al., 2005; Badawy, 2017). The first reaction can be catalyzed by TDO in the liver or by IDO1 in peripheral tissues, but the subsequent steps of kynurenine degradation mostly occur in the liver and the kidney (Terakata et al., 2013; Badawy, 2017).

Kynurenine is mainly oxidized by KMO (Kynurenine 3-monooxygenase) to 3-hydroxykynurenine, which is subsequently hydrolyzed by KYNU (Kynureninase) to 3-hydroxyanthranilate and alanine (Voet et al., 2005; Badawy, 2017). Further, HAAO (3-hydroxy-anthranilate 3,4-dioxygenase) catalyzes the oxidative breakdown of the 3-hydroxyanthranilate aromatic ring to produce 2-amino-3-carboxymuconate-6-semialdhyde, which is spontaneously converted into quinolinate. The latter can be metabolized by QPRT (Quinolinate phosphoribosyl transferase) in nicotinate mononucleotide, a precursor of NAD/NADP(H). Alternatively, substrate degradation can continue through oxidation and decarboxylation into acetoacetate. Kynurenine and 3-hydroxykynurenine can also be transaminated by KAT1-4 (Kynurenine aminotransferase 1–4) into kynurenic acid and xanthurenic acid, respectively (Badawy, 2017). However, transamination occurs less frequently than oxidation, because kynurenine aminotransferases exhibit a high K_m_ for both substrates in comparison with KMO and KYNU. Therefore, this alternative pathway becomes more substantial with kynurenine accumulation due to intense or extra-hepatic tryptophan degradation (Terakata et al., 2013; Badawy, 2017).

In summary, tryptophan degradation through the kynurenine pathway is mediated by TDO and IDO1. Although these enzymes are functionally homologous, their physicochemical properties are completely different (Table 1). They share only a low percentage of amino acid sequence identity and exhibit different conformations and affinities for substrates. Moreover, their genes are expressed in different tissues, which illustrates their different physiological functions.

**TABLE 1:** Comparison of IDO1 and TDO features.

	**TDO**	**IDO1**
Protein structure	Homotetramer 4 × 406 amino acids, 192 kDa	Monomer 403 amino acids, 45 kDa
Protein phylogeny	Well conserved	Poorly conserved
Human/Mouse identity	89%	69%
Substrate specificity	L-tryptophan	L-tryptophan, D-tryptophan, 5-hydroxytryptophan, tryptamine, serotonin
Kinetic parameters	Km = 190 µM^a^,^b^	Km = 20.9 µM^b^,^c^
	kcat = 2.1/s^a^,^b^	kcat = 2.97/s^b^,^c^
Tissue distribution	Liver (hepatocytes)	Placenta and lung (endothelial cells); Lymphoïd organs and intestine (dendritic cells); Urogenital organs (epithelial cells); Inflamed tissues
Regulation of gene expression	Upregulation by glucocorticoïds	Induction by IFNγ (TNFα and LPS)
Main functions	Maintenance of tryptophanemia; Synthesis of NAD/NADP(H)	Immunotolerance; Inhibition of pathogen growth
Deficiency	Hypertryptophanemia; Increased neurogenesis; Reduced anxiety; Increased blood and brain serotonin; Hypersensibility to endotoxaemia; Improved rejection of IDO-positive tumors	No major phenotypic alterations; Increased sensitivity to acute inflammation

a Reference: Batabyal and Yeh, 2007. Kinetics parameters reported for TDO were measured in enzymatic assays with stabilized recombinant enzyme. They may not reflect TDO activity in the liver in which the enzyme dissociates into inactive monomers in low tryptophan concentrations (Klaessens et al., 2021).

b The exact value of these parameters should be taken with caution, as they are based on *in vitro* enzymatic assays with recombinant enzymes. Ascorbic acid, with or without methylene blue, is used in these assays to prevent heme oxidation. The physiological reducing system may be different, potentially impacting kinetic parameters.

c Reference: Pantouris et al., 2014.

### 5.2 Tryptophan 2,3-dioxygenase

#### 5.2.1 TDO, main regulator of blood tryptophanemia

Among all actors involved in tryptophan homeostasis, TDO plays a leading role by assuming alone more than 90% of its catabolism (Kanai et al., 2009). Strongly expressed in the liver, TDO is situated at strategic crossroads to degrade the excess of dietary tryptophan coming from the portal vein and to filter the systemic tryptophan from the hepatic artery (Kanai et al., 2009; Hoffmann et al., 2020). Tryptophan degradation by TDO is required to contain tryptophanemia at 60 µM. Indeed, TDO-deficient humans and mice have plasmatic tryptophan concentrations 8 to 9-fold higher than that of healthy humans and wild-type mice (Ferreira et al., 2017 and Kanai et al., 2009, respectively). This increase is directly related to dietary tryptophan accumulation. Indeed, Maeta and colleagues showed that giving a low tryptophan diet to TDO-KO mice (0.06 vs. 0.17%) reduced their tryptophanemia to normal values (Maeta et al., 2013). Blood tryptophan accumulation in TDO-deficient mice induced an increase in serotonin synthesis, which is usually limited by the systemic tryptophan concentration (Kanai et al., 2009; Maeta et al., 2013). This could explain why TDO-KO mice exhibit less anxiety and more neurogenesis than wild-type controls. Blood tryptophan excess also increases the metabolism of tryptophan in kynurenine by IDO1 in peripheral tissues. As extra-hepatic tissues do not express kynurenine-degrading enzymes, this increases the circulating kynurenine concentration (Schramme et al., 2020). Except for this, no major alterations were related to TDO dysfunction. However, authors reporting the TDO-KO mouse phenotype were mainly focused on behavior and psychological diseases. In line with these observations, the TDO-deficient patient described by Ferreira and colleagues exhibited also an increase in blood serotonin concentration as a consequence of hypertryptophanemia (Ferreira et al., 2017). Medical checkup did not show any other obvious clinical symptoms. Nevertheless, her hypertryptophanemia was diagnosed at birth and partially corrected from childhood with a low tryptophan diet. Future investigations are required to understand the metabolic consequences of TDO inactivation.

Consistent with the key role of hepatic TDO in tryptophan homeostasis, a defect in tryptophan transport into hepatocytes can also unbalance systemic tryptophan homeostasis. Ingested aromatic amino acids are passively transported from blood into hepatocytes by SLC16A10 according to the concentration gradient. Mariotta and colleagues showed that SLC16A10 deficiency leads to tryptophan, phenylalanine and tyrosine accumulation in the blood (Mariotta et al., 2012). SLC16A10-KO mice have plasmatic tryptophan concentrations 3-fold higher than wild-type mice. This increase is less pronounced than that observed in TDO-deficient subjects. Such a difference could be explained by the contribution of other hepatic amino-acid transporters or the decrease of tryptophan absorption and reabsorption, as SLC16A10 also mediates the basolateral transport of aromatic amino acids in epithelial cells of the intestine and kidney. Indeed, Mariotta and co-workers showed that SLC16A10-deficient mice accumulated more radiolabeled phenylalanine in enterocytes of the small intestine and epithelial cells of renal tubules than wild-type mice (Mariotta et al., 2012). Accordingly, these mice showed a severe aromatic aminoaciduria exacerbated by high protein diet. Except for aromatic amino-acid blood accumulation and urinary losses, no phenotypic alterations were found in SLC16A10-deficient mice.

#### 5.2.2 TDO structure and physicochemical properties

**FIGURE 5:**
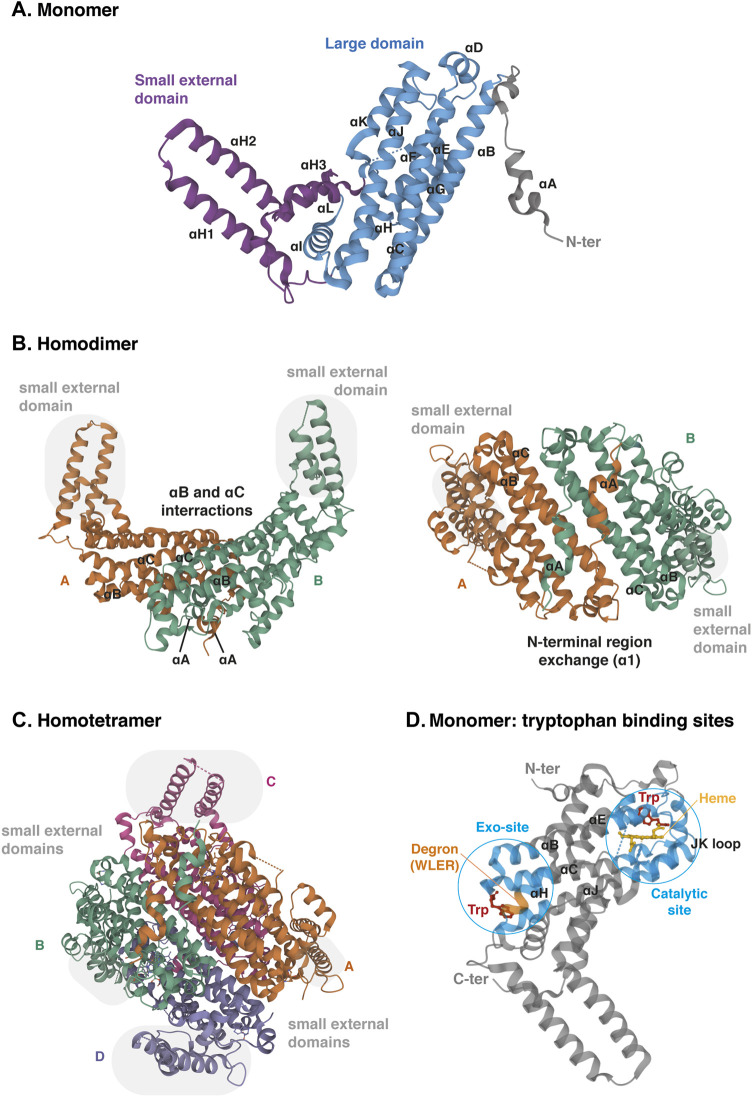
Crystal structure of human TDO (apo protein). **(A)** Monomeric conformation of TDO illustrating the secondary structure comprising fifteen α-helices distributed in three parts: the large central domain (blue), the small external domain (purple) and the N-terminal region (grey). α-Helices are labeled αA- αL according to the TDO structure described by Lewis-Ballester and colleagues (Lewis-Ballester et al., 2016). **(B)** Homodimeric conformation of TDO in two orientations. Monomers are distinguished by two colors. TDO monomers are associated in pairs through the interactions of their α-helices B and C of the large domains and through the exchange of their α-helices A of the N-terminal regions. Grey highlight shows the small external domains not involved in the interaction of monomers A and B. **(C)** Homotetrameric conformation of TDO. Monomers are distinguished by four colors. Dimers are clamped together through the interaction of their α-helices J. Dimer interaction is stabilized by the exchange of the small external domains (grey highlight) grafted on the opposite large domains (A with C and B with D, and vice versa). **(D)** Monomeric conformation of TDO illustrating the two tryptophan-binding sites. The TDO catalytic site (blue, right) is surrounded by the bundle from helices αB, αC, αJ, αE and αH. The heme cofactor (yellow) is bound by the proximal His328 from αJ. The TDO exo-site (blue, left) is also surrounded by the four-helical bundle from αB, αC, αJ, αE and αH, opposite the catalytic site. The orange color inset indicates the four-amino acid degron masked by tryptophan, triggering TDO degradation in low tryptophanemia (Klaessens et al., 2021). The 3D structure of TDO was modelized with Mol* Viewer from the crystal structure of TDO described by Lewis-Ballester et al. (2016) (PDB file ID: 5TIA).

The TDO protein is encoded by gene *TDO2*, which is located on chromosome 4q32. Its sequence has been well conserved during evolution since the human protein shares 89% of amino acid sequence identity and 95% of similarity with the mouse protein (protein BLAST database) (Table 1). TDO, which was initially called tryptophan pyrrolase, is a cytosolic heme dioxygenase that requires a Fe^2+^ ion to ensure the oxidative cleavage of the tryptophan indole group (Thackray et al., 2008). The crystal structure of human TDO was first reported in apo form, without heme cofactors and substrates (Meng et al., 2014) and then in holo form, with tryptophan (Lewis-Ballester et al., 2016). Both tridimensional structures are similar, but tryptophan binding sites and catalytic mechanisms were better defined by Lewis-Ballester and coworkers. Human TDO (hTDO) possesses a homotetrameric conformation (192 kDa) containing four catalytic sites (Meng et al., 2014; Lewis-Ballester et al., 2016). Each monomer comprises fifteen α-helices, but no β-strands and is organized in three regions: a large domain in the center, a small external domain and a N-terminal region (Figure 5A). The scaffold can be described as a dimer of dimers. First, monomers are associated in pairs through the interaction of αB and αC. In addition, monomers swap their N-terminal α-helix (αA) to form the active site of the other (Figure 5B). Secondly, the two dimers are clamped together through the interaction of αJ, to form the tetramer core. Additionally, the two dimers exchange their small external domains, which are grafted on the opposite active sites (Figure 5C). As a consequence, TDO is only active in its oligomeric conformation.

Each TDO subunit comprises an independent catalytic site (Figure 5D). The three α-helices forming the tetramer interface (αB, αC and αJ) and the long helix formed by the combination of αE and αH produce a four-helical bundle (Lewis-Ballester et al., 2016). This structure shapes a hydrophobic cavity in which the heme group is anchored, bound to the proximal His328 from the C-terminal extremity of αJ. In the distal side of the heme, the L-tryptophan substrate establishes a hydrogen bound with His76 from αB and an ionic interaction with dioxygen. The active site is then covered by the loop linking αJ and αK through a highly conserved GTGG amino-acid motif, whose sequence is identical in TDO of mice, *drosophila melanogaster* and Xanthomonas campestris (proteobacteria). TDO is particularly specific for L-tryptophan, and is unable to metabolize similar molecules, such as D-tryptophan or serotonin (Thackray et al., 2008).

In addition to the active site, each TDO subunit comprises a second tryptophan-binding site in the large domain, far away from the heme (Figure 5D) (Lewis-Ballester et al., 2016). This noncatalytic exo-site is located at the other extremity of the four-helical bundle shaping the active site. The L-tryptophan side chain is anchored in a hydrophobic area between Trp208 and Pro213 of αH. The carboxylate group of tryptophan interacts by an ionic bound with Arg211. Additionally, the ammonium group of tryptophan is stabilized by the main chain carboxyl group of Arg103 and the side chain of the Glu105 from αB and αC, respectively. These residues have been highly conserved among TDO proteins from other organisms, but not in ortholog proteins such as IDO1. Lewis-Ballester and colleagues initially suggested that the binding of tryptophan to these exo-sites could stabilize TDO by reducing its degradation by the ubiquitin proteasome system. Although they proposed that binding of tryptophan in exo-sites regulates TDO half-life, they did not describe the molecular mechanism, nor the physiological significance of TDO stability.

#### 5.2.3 Regulation of TDO activity

**FIGURE 6:**
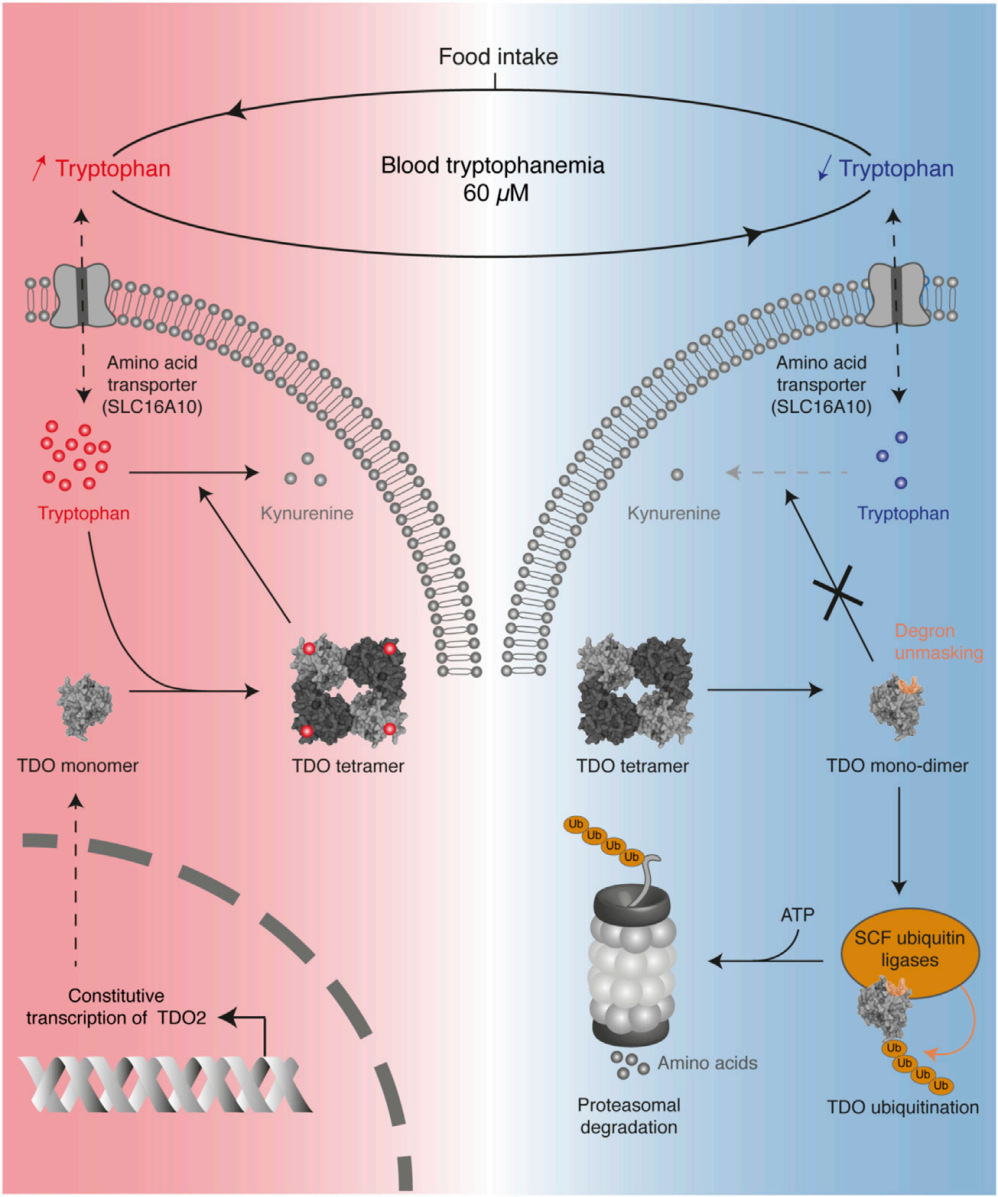
Overview of the mechanism regulating TDO stability in the liver to control blood tryptophanemia. Protein intake increases the blood level of tryptophan, which accumulates in hepatocytes (red color). High tryptophan levels stabilize the tetrameric conformation of TDO through the binding of tryptophan in four exosites, thereby stimulating tryptophan catabolism. When tryptophan becomes scarce (blue color), the absence of tryptophan binding the exosites destabilizes the tetrameric structure of TDO in inactive monomers and dimers. Absence of tryptophan unmasks a degron in the exosites, which triggers TDO ubiquitination by SKP1-CUL1-F-box complexes. This finally induces TDO degradation by the proteasome, thereby stopping tryptophan catabolism.

Recently, we reported that systemic tryptophan homeostasis is mainly controlled by the TDO post-translational stability, which is regulated by the binding of tryptophan in the exo-sites (Klaessens et al., 2021). We showed that the TDO structure acts as a tryptophan sensor, adapting tryptophan catabolism to dietary supply (Figure 6). In the post-prandial period, high tryptophan availability increases the TDO protein level in the liver, resulting in rapid normalization of tryptophanemia. Structurally, TDO binds tryptophan in the four heme-containing catalytic sites and the four exo-sites. The binding of tryptophan in exo-sites increases TDO half-life and stabilizes the homo-tetrameric structure of TDO, boosting tryptophan catabolism. Once tryptophan concentration is low, tryptophan leaves the exosites, resulting in TDO dissociation into inactive monomers and dimers. These changes unmask a 4-amino acid degron whose sequence WLER_208-211_ was initially masked by tryptophan in exosites (Figures 5D, 6). Once accessible, the degron triggers TDO ubiquitination by E3 SKP1-CUL1-F-box, resulting in rapid TDO degradation by the proteasome, irrevocably interrupting tryptophan catabolism. TDO stability is the critical mechanism limiting tryptophan catabolism below the physiological concentration of 60 µM. Indeed, we showed in mice that permanent stabilization of hepatic TDO with alpha-methyl-tryptophan, a ligand binding the exo-sites but not the catalytic sites, decreases blood tryptophan levels to 20 µM. It must be emphasized that tryptophan homeostasis is regulated independently of the transcription of *TDO2* since its mRNA level remains constant regardless of tryptophan concentration. Such a post-translational mechanism in which TDO is directly stabilized and activated by tryptophan allows a quick and fine regulation of blood tryptophanemia. Our results are consistent with the old observations of Knox and colleagues, showing that tryptophan enhances TDO activity in rat liver homogenates (Civen and Knox, 1959). The group of Schimke later showed by radioactive leucine incorporation that tryptophan delayed the degradation of prelabeled TDO (Schimke et al., 1965).

Interestingly, the human case of hypertryptophanemia due to TDO deficiency described by Ferreira and colleagues was a compound heterozygote of two TDO variants (Ferreira et al., 2017). The first allele with a c.491 duplication resulted in a frame shift, which introduced a premature stop codon and generated a catalytically inactive protein truncated at 43% of its normal length. The second allele c.324 G>C generated a complete protein with a point mutation of highly conserved Met108 in Ile108. This residue is structurally close to Arg103, Glu105 and Trp208, which bind tryptophan in the exo-sites, as described above. This prompted the authors to investigate the binding of tryptophan in TDO exo-sites, which was 100-fold reduced in the variant p.Met108Ile. Although the authors did not explore the consequences *in vivo*, this suggests that the point mutation p.Met108Ile accelerated TDO degradation, explaining why the liver was not able to normalize tryptophanemia.

Of note, the previous papers describing TDO activity *in vitro* did not observe the TDO destabilization in low tryptophan level occurring *in vivo*, probably due to unphysiological experimental conditions (Batabyal and Yeh, 2007; Meng et al., 2014). Therefore, the K_m_ and k_cat_ values of TDO reported in enzymatic assays may not reflect the enzyme activity in the liver (Table 1).

Expression of *TDO2* in hepatocytes is constitutive. Nevertheless, its transcription in the liver is upregulated by glucocorticoids (Civen and Knox, 1959; Danesch et al., 1983). Studies revealed that injection of hydrocortisone or dexamethasone in rat increased the mRNA level and the activity of TDO, which were assessed in liver homogenates. This transcriptional regulation is in line with the presence of two separated glucocorticoid responsive elements in the rat *TDO2* promoter (Danesch et al., 1987). Accordingly, induction of TDO activity by corticosterone in rats was shown to reduce tryptophan concentration in serum and liver (Badawy, 1981).

#### 5.2.4 Immunoregulatory functions of TDO

Tryptophan catabolism plays a key role in immune tolerance by decreasing the tryptophan concentration and accumulating tryptophan catabolites, both inducing T-cell inhibition. These immunosuppressive mechanisms have been largely described for IDO1, whose expression is induced by pro-inflammatory stimuli. Recent studies showed that TDO mediates also immunosuppressive effects, but physiologically different from IDO1. Hepatic TDO maintains the systemic tryptophan concentration around 60 µM, which is much higher than the amino acid level of 1 µM causing inhibition of T-cell proliferation (Munn et al., 1999). As a consequence, the immunosuppressive effects of hepatic TDO activity are mainly mediated by the production of kynurenine and derivatives, rather than by tryptophan depletion. By containing tryptophanemia, TDO can nevertheless contribute to tryptophan depletion mediated by IDO1 in peripheral tissues. Two recent discoveries highlight the systemic role of TDO in immune tolerance, arguing that TDO-deficient subjects could develop overactive immune responses.

In 2014, Bessede and colleagues showed that TDO inactivation increased susceptibility to endotoxemia (Bessede et al., 2014). Indeed, TDO-KO mice or normal mice treated with TDO inhibitor 680C91 died after injection of an LPS dose that was only sublethal for wild-type controls. Interestingly, AHR-KO mice exhibited the same susceptibility to LPS than TDO-KO mice, suggesting an immunosuppressive effect of tryptophan catabolites against endotoxemia mediated by AHR. Convergently, LPS administration increased blood kynurenine concentration threefold in wild-type mice, but not in TDO-KO mice. This report suggested that LPS administration upregulated hepatic TDO, resulting in accumulation of plasmatic kynurenine, which downregulated early inflammatory gene expression through AHR activation. The impact of tryptophan catabolism by TDO on the peripheral tolerance and prevention of septic shock underlines its key role in immunity, which should be studied with the aim to prevent autoimmune diseases.

In 2020, we found that a high systemic concentration of tryptophan improved anti-tumor immune responses (Schramme et al., 2020). Indeed, we showed that anti-PD1 and anti-CTLA4 checkpoint inhibitors induced the immune rejection of transplanted MC38 tumors in TDO-KO mice, while the same tumors escaped the immune response in wild-type mice. This effect was related to the increased circulating tryptophan in TDO-KO mice (450 µM), as the normalization of tryptophanemia with a low tryptophan diet abolished tumor rejection in TDO-KO mice. Transcriptomic analysis revealed that MC38 tumors expressed *IDO1*, which depleted the local tryptophan concentration and limited the efficacy of immune checkpoint inhibitors. High systemic concentration of tryptophan presumably overcame IDO1 capacity to decrease the local tryptophan concentration to a level that was sufficiently low to induce effective immunosuppression. Consistent with this hypothesis, IDO1 inhibitor epacadostat improved the efficacy of the anti-PD1 immune checkpoint inhibitor in wild-type mice, but not in TDO-KO mice. Together, these results support the notion that the circulating tryptophan concentration controlled by TDO influences the peripheral tolerance linked to IDO1-mediated tryptophan depletion.

#### 5.2.5 TDO, actor of tumor immune escape

To escape destruction by immune cells, tumor cells often hijack immunosuppressive mechanisms. In this way, certain tumors acquire the capacity to degrade tryptophan by expressing TDO or IDO1. Whole transcriptome data analysis of human healthy and tumoral tissues revealed that *TDO2* is highly expressed in the liver, while its expression in other normal tissues is close to zero (van Baren and Van den Eynde, 2015b). As expected from its hepatic origin, hepatocarcinoma presents high levels of *TDO2* transcripts. In contrast with their normal counterparts, tumors of various other histological types also express TDO. This underpinned the results obtained by RT-PCR on tumor samples, which revealed that TDO is expressed in numerous human tumors, especially in hepatocellular carcinoma, bladder carcinoma, melanoma and glioblastoma, as well as in tumor lines, including glioblastoma, colorectal, head and neck and bladder carcinoma cell lines (Opitz et al., 2011; Pilotte et al., 2012). To identify the type of cells expressing TDO in tumor samples, we recently developed TDO-specific antibodies and detected the protein by immunohistochemistry in human tumor tissues (Hoffmann et al., 2020). In line with transcriptomic studies, TDO was found in a majority of these tumor samples. In hepatocarcinomas, TDO was expressed in tumor cells themselves, while in other tumors it was mainly present in pericytes of blood vessels, but only in a minority of tumor cells.

In 2012, we provided evidence that constitutive expression of TDO in tumors prevented their immune rejection (Pilotte et al., 2012). As no mouse tumor expressing TDO has been identified, we transfected TDO in mastocytoma cell line P815B, which presents the MAGE-type tumor rejection antigen P1A. Mice immunized with P1A rejected the transplanted P815B tumor cells, while the second group of mice challenged with TDO-expressing P815B cells did not. Further investigations revealed that immunized mice contained detectable amounts of anti-P1A specific CTL. Their number increased 3-fold after the challenge with P815B control cells, while this increase was moderate with the P815B tumors expressing TDO, suggesting that TDO inhibited T-cell proliferation. These effects were due to tryptophan catabolism, as TDO inhibitor LM10 abolished tumor progression. These results provided the proof of concept that TDO inhibition can combat tumor-induced immunosuppression and be used for immunotherapy.

Convergent results obtained by Opitz and colleagues revealed that tumors expressing TDO degrade tryptophan in kynurenine, which suppresses an anti-tumor immune response through the AHR axis (Opitz et al., 2011). First, they showed that TDO-expressing U87 glioma cells cultured with allogeneic peripheral blood mononuclear cells (PBMC) suppressed T-cell proliferation, an effect that was abolished by TDO knockdown. This effect was related to tryptophan catabolites, as addition of kynurenine alone prevented the restoration of T-cell proliferation triggered by TDO knockdown. *In vivo* experiments showed that murine GL261 glioma cells transfected with TDO grew faster than control cells, which was associated with an immune escape demonstrated by the decreased activity of tumor-infiltrating lymphocytes (TILs). The authors analyzed by microarray the transcriptome of kynurenine-treated glioma and found an AHR signature, characterized by an upregulation of AHR-target genes such as CYP1B1 (Cytochrome P450 family 1 subfamily B member 1) and TIPARP (TCDD-inducible poly-ADP-ribose polymerase). Subsequent analysis revealed that autocrine kynurenine in glioma cells activated the AHR pathway, resulting in an increase of cell survival and motility. Interestingly, the proliferation of TDO-expressing GL261 tumors was attenuated in AHR-KO mice in comparison with wild-type mice. This revealed that AHR from host cells also influenced the development of tumors, through the released kynurenine. TDO expression decreased the infiltration of tumors with leukocytes LCA^+^ (Leukocyte common antigen) and CD8^+^ in wild-type mice, but not in AHR-KO mice which exhibited a number of TILs similar to TDO-deficient tumors. Together, their results suggest that tryptophan catabolism by TDO in tumors generates kynurenine, which activates the AHR pathway in adjacent cells through a paracrine fashion, contributing to tumor immune escape. Altogether, these findings have encouraged the development of inhibitors targeting TDO (Dolusic et al., 2011; Platten et al., 2019; Kozlova et al., 2021).

### 5.3 Indoleamine 2,3-dioxygenase

#### 5.3.1 IDO1, a peripheral enzyme depleting tryptophan locally

In contrast to TDO, IDO1 weakly influences blood tryptophanemia, but it ensures local tryptophan degradation in peripheral tissues. It accounts for only 5% of overall tryptophan catabolism, but becomes more substantial under immune activation (Badawy, 2017). IDO1 is constitutively expressed in placental and lung endothelial cells, mature dendritic cells and urogenital epithelial cells. It is also induced in many cells during inflammation, as IDO1 is one of the genes whose transcription is most upregulated by interferon gamma (Munn et al., 1998; Theate et al., 2015). The enzyme plays a leading role in peripheral tolerance by depleting tryptophan concentration to submicromolar ranges. Tryptophan depletion and kynurenine accumulation both create an immunosuppressive environment inhibiting T lymphocytes. In line with IDO1 peripheral functions, IDO1-KO mice exhibit a systemic concentration of tryptophan close to wild-type mice (Schramme et al., 2020). As extrahepatic tissues do not express kynurenine-degrading enzymes, kynurenine generated by IDO1 is released in the extracellular medium, before being filtered by the liver. Consequently, IDO1-deficient mice have a low circulating kynurenine concentration in comparison with normal subjects, as opposed to TDO-deficient mice (Schramme et al., 2020). No major phenotypic alterations have been observed in IDO1-deficient mice, but they present an increased sensitivity to induction of acute inflammation (Chang et al., 2011).

#### 5.3.2 IDO1 structure and physicochemical properties

**FIGURE 7:**
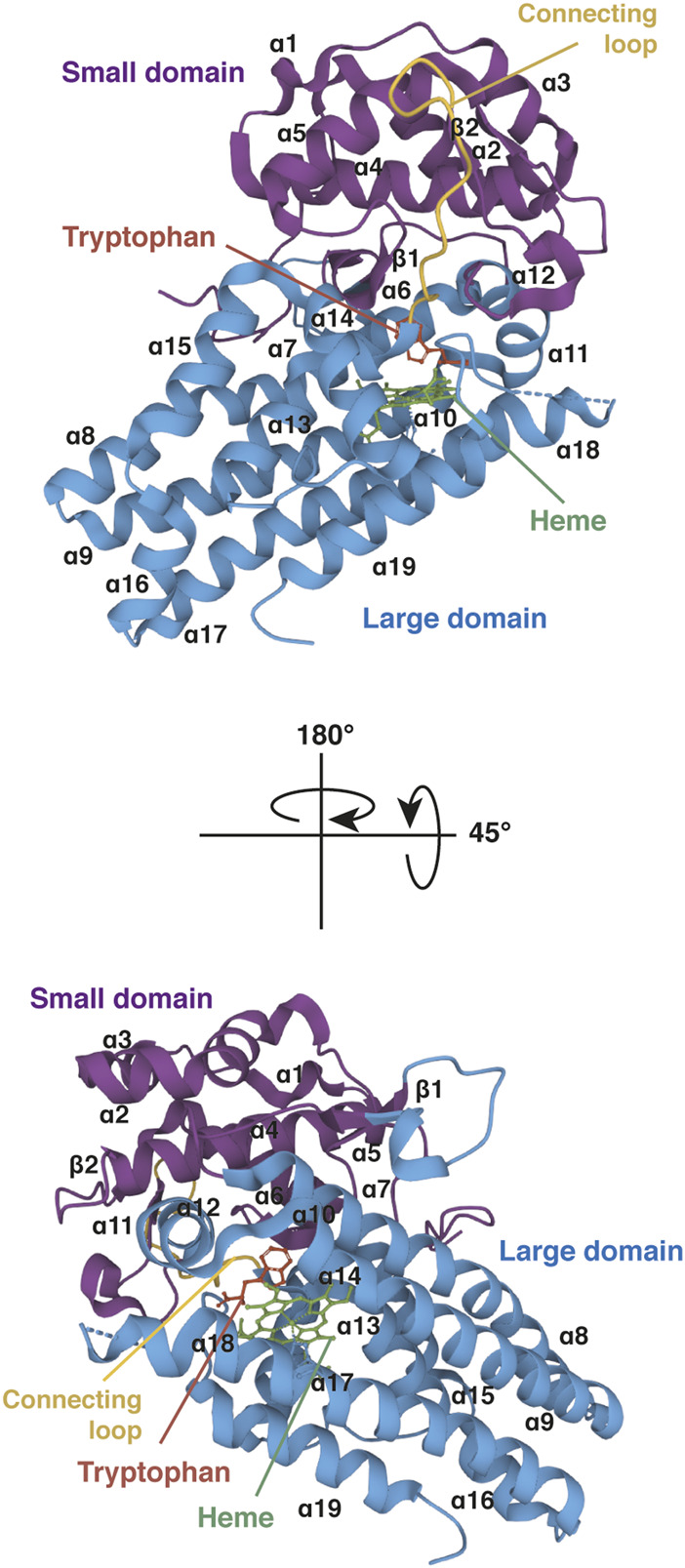
Crystal structure of human IDO1 (holo protein). Structure of monomeric IDO1 complexed with tryptophan (red) and the heme cofactor (green) in two orientations. The secondary structure of IDO1 comprises nineteen α-helices and two β-strands assembled in a large domain (blue) and a small domain (purple) arranged on either side of the heme binding site. Both domains are connected together by a long loop (yellow). The heme binding site is surrounded at the proximal side by α-helices 7, 9, 17, and 19 of the large domain and at the distal side by the small domain and the connecting loop. The proximal side refers to the proximal histidine forming a coordinate covalent bound with the iron ion at the center of the heme group, and the distal side to the other side of the heme where iron binds dioxygen. The tryptophan-binding site is shaped by α-helices 11, 12, and 14 of the large domain and the connecting loop, close to the distal side of the heme. The 3D structure of IDO1 was modelized with Mol* Viewer from the crystal structure of IDO1 described by Luo et al. (2018) (PDB file ID: 6E46).

Gene *IDO1* is located on the p11 region of chromosome 8. The *IDO1* sequence has been poorly conserved during evolution since human protein shares only 63% amino acid identity and 77% similarity with the mouse protein (protein BLAST database) (Table1). Although IDO1 and TDO are heme dioxygenases catalyzing exactly the same reaction, their sequences are fundamentally different and do not exhibit common origins. The crystal structure of human IDO1 was first reported in holo form with IDO1 inhibitors (Sugimoto et al., 2006) and then with tryptophan (Luo et al., 2018). In contrast to TDO, IDO1 exhibits a monomeric conformation (45 kDa), which contains a unique catalytic site including a heme cofactor (Figure 7). IDO1 is folded in two domains arranged on either side of the heme binding site: a large domain composed of thirteen α-helices and two 3_10_ helices and a small domain composed of six α-helices, two β-strands and three 3_10_ helices (Sugimoto et al., 2006). The large domain shapes a hydrophobic cavity with four long alpha helices (7, 9, 17, and 19) enclosing the proximal heme side. Surrounding the heme cofactor, the two domains interact together through a large area delimited by three alpha helices of the large domain (11, 12, and 14) and a long connecting loop. The heme-binding site is closed by the small domain and the connecting loop, which cover the distal heme side. The tryptophan binding site is shaped by the connecting loop and the alpha helices of the large domain (11, 12, and 14), close to the distal side of the heme where oxygen cleaves the tryptophan indole group. In contrast to TDO, IDO1 is not very specific for L-tryptophan and can oxidize a broad range of analogous molecules, including D-tryptophan, 5-hydroxytryptophan and serotonin (Badawy, 2017). IDO1 exhibits a low K_m_ of 21 µM and a high catalytic efficiency (k_cat_/K_m_). This allows depleting tryptophan in peripheral tissues below normal tryptophanemia (60 ± 15 µM) (Table 1) (Pantouris et al., 2014). In contrast, TDO cannot degrade tryptophan at low concentrations as the enzyme dissociates into inactive monomers below normal tryptophanemia (Klaessens et al., 2021).

#### 5.3.3 Regulation of IDO1 activity

IDO1 is not or weakly expressed in the majority of human tissues, but its expression is triggered by pro-inflammatory stimuli. Many studies showed that IFNγ, a cytokine released by activated T cells, induces IDO1 expression and activity in human cell lines (Pfefferkorn et al., 1986b; Carlin et al., 1987). This regulation was confirmed *in vivo* in human patients treated with recombinant IFNγ, which exhibited a moderate decrease in plasmatic tryptophan concentration and an accumulation of kynurenine in urine (Byrne et al., 1986a). Subsequent studies revealed that IDO1 expression is regulated by IFN-stimulated response elements (ISRE) and gamma activation sequences (GAS) in the *IDO1* promoter (Dai and Gupta, 1990; Hassanain et al., 1993). Other pro-inflammatory stimuli can upregulate *IDO1* transcription, such as TNFα and IL-1. Nevertheless, these factors play a secondary role in IDO1 activation and require IFNγ to induce degradation of tryptophan (Babcock and Carlin, 2000). Nitric Oxyde (NO), which can be produced by nitric oxide synthases during inflammation, was shown to inhibit IDO1 by binding the heme active site (Thomas et al., 1994; Thomas et al., 2007).

#### 5.3.4 Immune functions of IDO1

Tryptophan catabolism by IDO1 was first considered as an adaptive host immune mechanism starving intracellular pathogens. Indeed, Pfefferkon and colleagues showed that human recombinant IFNγ inhibited the growth of Toxoplasma gondii cultured in fibroblasts (Pfefferkorn and Guyre, 1984). This effect was later associated to the critical decrease in tryptophan concentration due to IDO1 induction (Pfefferkorn et al., 1986b). Subsequent publications corroborated that tryptophan deprivation mediated by IDO1 represses the proliferation of intracellular pathogens auxotrophic for tryptophan, such as *Chlamydia* and *Coxiella*, arguing that IDO1-deficient subjects could be more sensitive to infections (Byrne et al., 1986b; Ganesan and Roy, 2019).

At the end of the nineties, tryptophan catabolism by IDO1 emerged as a powerful immunosuppressive mechanism, involved in the feedback control of the immune response and in peripheral tolerance. In 1999, Munn and colleagues noticed that macrophages differentiated under macrophage-colony stimulating factors (MCSF) and cocultured with activated T lymphocytes acquired the capacity to inhibit the activation of naive T lymphocytes (Munn et al., 1999). HPLC and transcriptomic analysis revealed that IFNγ from activated T cells induced IDO1 expression in macrophages, resulting in rapid tryptophan depletion inhibiting T-lymphocyte activation. Naive T cells activated in tryptophan-free medium were able to synthesize proteins, including IL-2 and IFNγ, meaning that translation was not inhibited. However, tryptophan deprivation in early phases of activation blocked the cell cycle of T cells in phase G1, interrupting their proliferation. A dose response experiment showed that proliferation of T lymphocytes was compromised only in low tryptophan concentrations, under 1 µM. Once the cells stop dividing, restoring the concentration of tryptophan was not sufficient to restart the cell cycle. T cells were not blocked forever, but needed a second cycle of activation in the presence of tryptophan to proliferate again. This feedback mechanism triggered by IFNγ obviously helps to prevent an overactive immune response (Theate et al., 2015).

In parallel, the same group published that tryptophan catabolism mediated by IDO1 in placenta protects the fetus against immune rejection (Munn et al., 1998). As IDO1 is highly expressed in syncytiotrophoblast cells at 7.5–9.5 days post-coitus (dpc), they postulated that tryptophan catabolism could inhibit maternal T cells. Female mice mated with allogeneic or syngeneic male were treated or not with IDO1 inhibitor 1-methyl-tryptophan from 4.5 dpc. Before IDO1 expression, at day 6.5, the mice from all groups carried the same number of embryos and no clinical symptoms were detected. In contrast, at day 7.5, the number of allogeneic embryos was significantly lower in mice treated with the IDO1 inhibitor; this reduced number was associated with a severe bleeding visible in residual embryos. This became worse with the progression of pregnancy to the extent that all embryos showed signs of inflammation between days 8.5 and 9.5 and were rejected before day 9.5. No clinical manifestations were detected in mice carrying syngeneic embryos treated with IDO1 inhibitors, proving that rejection was not triggered by the IDO1 inhibitor itself and suggesting it was immune-mediated. This prompted the authors to investigate whether maternal lymphocytes were involved in the loss of allogeneic concepti by inactivating the RAG1 protein (Recombination activating gene 1) required for lymphocyte development. As expected, mouse infertility caused by IDO1 inhibition was abolished in immunodeficient RAG1-KO female mice carrying allogeneic embryos. Altogether, these results provided the first evidence that tryptophan catabolism by IDO1 contributes to peripheral tolerance *in vivo.*

The immunoregulatory functions of IDO1 were later studied in the context of immunopathologies. The group of Prendergast showed that loss of IDO1 increased the sensitivity to induction of acute inflammation (Chang et al., 2011). They showed that administration of complete Freund adjuvant, a mixture of lipids composed of *Mycobacterium tuberculosis* extracts known to activate IDO1 expression, induced severe pancreatitis in IDO1-KO mice in comparison with wild-type controls. The concept was then extended to another model of acute inflammation, supporting a major role of IDO1 in peripheral tolerance: IDO1 deficiency increased the incidence of acute colitis triggered by pro-inflammatory stimuli, which promoted, in combination with carcinogens, the development of colon carcinomas (Chang et al., 2011).

#### 5.3.5 IDO1, a key actor in tumor immune escape

Like TDO, IDO1 is expressed in numerous tumors in which it contributes to tumor immune escape. Whole transcriptome data and immunohistochemistry analysis of human healthy and tumoral tissues revealed that IDO1 expression, which is normally restricted to placental, lung and urogenital tissues, is detected in numerous tumors, especially in cervical, endometrial, lung and kidney carcinomas (Uyttenhove et al., 2003, van Baren and Van den Eynde, 2015b). This was corroborated by a large immunohistochemistry screening performed on various human tumors, which reported the presence of the IDO1 protein in 61% of samples (505/866), mainly in carcinomas (Theate et al., 2015). This study distinguished two profiles of tumor cells expressing IDO1. In some samples, IDO1-positive tumor cells were detected in inflamed areas rich in T lymphocytes. This distribution was similar to other proteins induced by IFNγ, suggesting an adaptive immune resistance mechanism. In other samples, tumor cells expressing IDO1 were observed without any sign of inflammation or T-cell infiltration. This constitutive expression was in line with that observed in tumor lines (Uyttenhove et al., 2003) and was subsequently found to be triggered by an autocrine loop of prostaglandin E2 secretion produced by COX2 (Cyclooxygenase 2) expressed as a result of oncogenic signaling (Hennequart et al., 2017).

In 2003, our group discovered that tryptophan catabolism by tumor lines expressing constitutively IDO1 prevented their immune rejection (Uyttenhove et al., 2003). Mice immunized against the P1A antigen completely rejected transplanted P815B tumor cells, while the second group of mice treated similarly with P815B cells expressing IDO1 did not. This was due to the ability of IDO1-expressing tumor cells to prevent their immune rejection, as both types of tumors progressed with the same kinetics in immunocompromised/irradiated mice. Immune protection conferred by IDO1 expression was not related to a loss of antigenicity, as both tumors were equally lysed *in vitro* by anti-P1A specific CTL. Additional analysis revealed that mice challenged with P815B tumors presented higher proportions of anti-P1A specific CTL, than mice challenged with IDO1-expressing P815B tumors, suggesting that IDO1 blocked T-lymphocyte proliferation. Pro-tumoral effects of IDO1 expression were abolished in mice treated with IDO1 inhibitor 1-methyl-tryptophan, proving the key role of tryptophan catabolism in tumor immune escape. Subsequent work in a different model revealed that inhibition of IDO1 constitutive expression in tumor cells with COX2 inhibitor celecoxib also promoted tumor immune rejection (Hennequart et al., 2017).

Convergent results were obtained by Zheng and colleagues with another mouse tumor model (Zheng et al., 2006). B16F10 melanoma cells expressing constitutively IDO1 were shown to inhibit the proliferation and induce the apoptosis of naive co-cultured T cells. These effects were abolished by silencing IDO1 expression with siRNA or inhibiting IDO1 with 1-methyl-tryptophan. In line with this *in vitro* analysis, B16F10 transplantable melanomas grew more slowly in mice treated with IDO1 inhibitors or IDO1-siRNA injected intratumorally, than in control mice. This was associated with an increased CD8^+^ T cell infiltration in IDO1-silenced tumors. Numerous subsequent studies related similar observations, which led to the notion that IDO1 tumor expression is a hallmark of cancer associated with a poor prognosis (Godin-Ethier et al., 2011). These studies prompted the development of pharmacological inhibitors of IDO1 (Prendergast et al., 2017; Platten et al., 2019). The first Phase III clinical trial of an IDO1 inhibitor administered with anti-PD-1 to melanoma patients failed to improve their survival (Long et al., 2019). However, several reasons can explain this negative outcome, such as an insufficient inhibition of IDO1 activity or an inadequate selection of patients whose tumors were not tested for IDO1 expression (Van den Eynde et al., 2020).

## 6 Tryptophan blood transport

Blood tryptophan, whose total concentration is around 60 µM, exists in equilibrium between a free form and an albumin-bound form, which accounts for 80–90% (McMenamy et al., 1961). Only the free-tryptophan is metabolically available for cells. However, tryptophan binding to albumin is a quickly reversible process, so that albumin rapidly buffers the variations of free-tryptophan concentration (Smith and Pogson, 1980). Equilibrium between the two forms is a labile parameter influenced by plasma albumin levels and disponibility. Increase of albumin content was shown to reduce the pool of plasma free tryptophan, resulting in a decrease of hepatic tryptophan uptake and catabolism. On the contrary, elevation of long-chain non-esterified fatty-acids, which compete for albumin binding, induces tryptophan release and degradation by hepatic TDO (Curzon and Knott, 1974; Smith and Pogson, 1980). This seems an important physiologic event for pregnancy, in which a high free-tryptophan level in plasma was associated with a decrease in albumin level and an elevation of fatty acids (Badawy, 1988).

## 7 Overall balance of tryptophanemia

**FIGURE 8:**
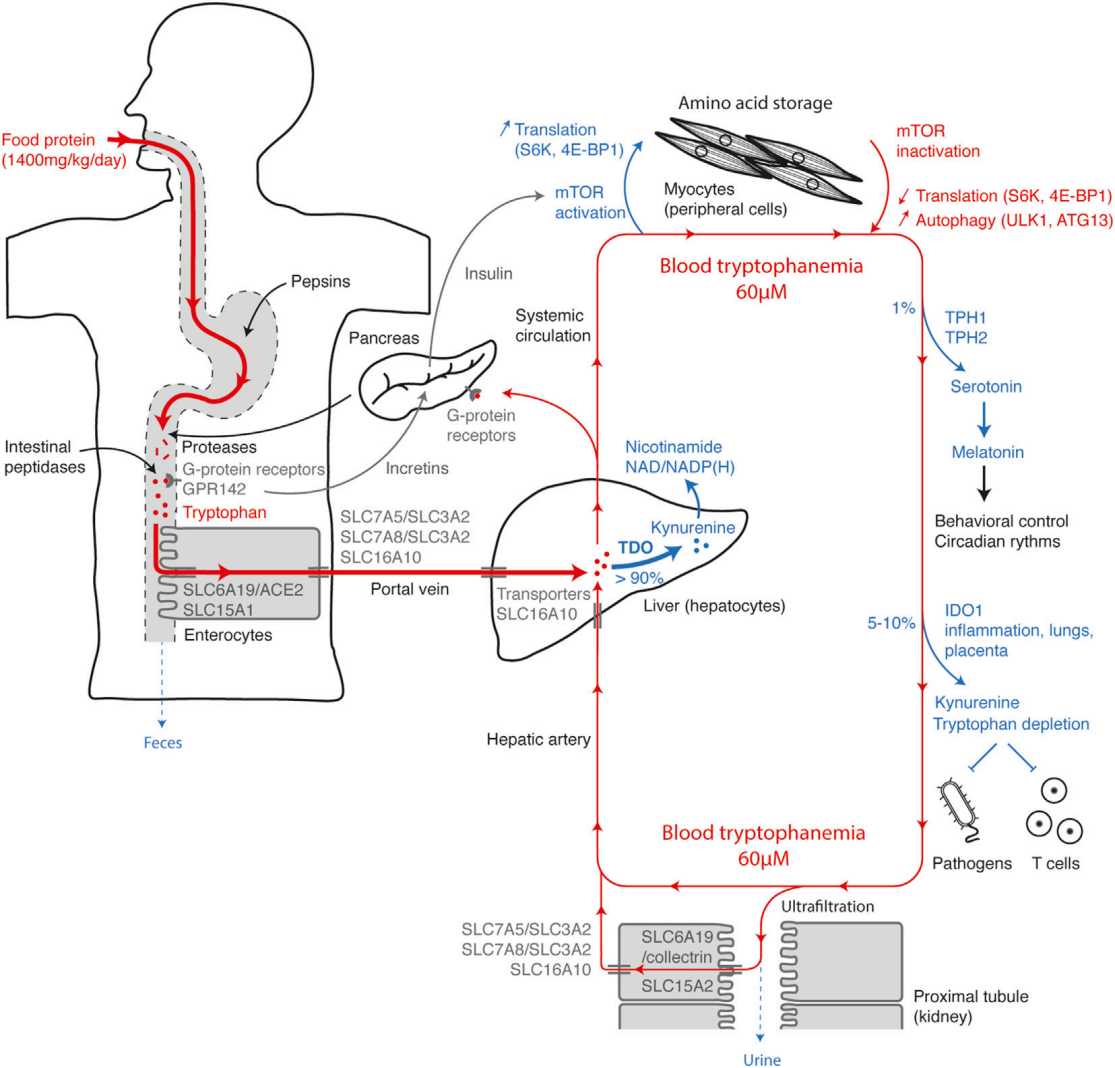
Tryptophan homeostasis overview. The figure represents the main checkpoints of tryptophan homeostasis from food intake to elimination. The steps increasing the systemic concentration of tryptophan are depicted in red and the steps decreasing tryptophan, in blue. Entry of tryptophan in the systemic circulation is first conditioned by intestinal absorption through polarized cells (left side), optimized to get a maximum of dietary tryptophan. Once absorbed, the tryptophan contained in the portal vein is filtered by the liver and mainly metabolized by TDO (center), before entering in the systemic circulation. High amino-acid levels and postprandial hormones activate the mTOR pathway in muscles and peripheral tissues (top), stimulating protein synthesis and amino-acid storage. When amino-acid levels are low, inactivation of mTOR slows down translation and induces protein autophagy, which contributes to amino-acid saving and release. A small percentage of tryptophan is metabolized in serotonin by TPH1/2 and in kynurenine by IDO1 in peripheral tissues (right side). Almost all the tryptophan present in the glomerular ultrafiltrate is actively reabsorbed by polarized cells of the proximal renal tubules (bottom). Excess circulating tryptophan is filtered by hepatocytes and degraded by TDO (center).

To discuss how each mechanism influences systemic tryptophan homeostasis, it is interesting to summarize the tryptophan movements from consumption to elimination (Figure 8). Tryptophan, whose daily supply is on average 12 mg/kg, is mainly contained in food proteins. Along the digestive tract, proteins are progressively digested into peptides and free amino acids by gastric pepsins, pancreatic proteases and intestinal peptidases. Dietary amino acids are directly detected along the taste buds and the gastrointestinal tract by GPCRs. Their activation helps to anticipate the amino-acid influx through the release of incretins that stimulate insulin secretion by pancreatic islets. Tryptophan is detected by broad-spectrum GPCRs, as well as by the tryptophan-specific sensor GPR142. Free tryptophan and small peptides are captured by enterocytes through the active apical transporters SLC6A19/ACE2 and SLC15A1. Tryptophan is next released in the portal vein through the basolateral transporters SLC7A5/SLC3A2, SLC7A8/SLC3A2, and SLC16A10. Blood of the portal vein is then filtered by hepatocytes into which tryptophan is transported by the passive transporter SLC16A10. More than 90% of the tryptophan is degraded into N-formylkynurenine by TDO in the liver, during the first hepatic passage or the blood filtration from the hepatic artery. This step, which represents the main checkpoint of tryptophanemia, is controlled by a TDO-intrinsic tryptophan-sensing mechanism governing TDO stability and activity. As hepatocytes express all the enzymes of the kynurenine pathway, tryptophan is directly metabolized to alanine and acetoacetate or quinolinic acid, the precursor of NAD/NADP(H). Unmetabolized tryptophan enters in the systemic circulation, before being distributed in peripheral tissues.

Postprandial increase in plasmatic amino-acid concentration is detected by nutrient-sensing GPCRs in β cells of Langerhans islets, which secrete insulin. The combined increase of amino acids and insulin activates the mTOR-signaling pathway in all tissues, promoting amino-acid storage by stimulating protein synthesis. Conversely, when amino acids are lacking, mTOR inactivation leads to autophagy through ULK1-ATG13 activation, releasing amino acids stored in proteins, especially in muscle proteins. In addition to protein synthesis, approximately 1% of dietary tryptophan is metabolized through TPH1/2 in serotonin and melatonin. Their synthesis is limited by the systemic concentration of tryptophan. 5–10% of tryptophan is degraded by IDO1 expressed in the placenta, lungs, urogenital tract and dendritic cells or induced by pro-inflammatory stimuli. Peripheral tryptophan depletion limits the growth of intracellular pathogens and contributes to the feedback control of the immune response. Amino acids and small peptides spontaneously cross the glomerular barrier. To limit losses, tryptophan and peptides are actively reabsorbed in the primary urine through SLC6A19/collectrin and SLC15A2 transporters, respectively, and then released in peritubular capillaries through the basolateral transporters SLC7A5/SLC3A2, SLC7A8/SLC3A2, and SLC16A10. Therefore, in healthy subjects, tryptophan elimination through the urinary tract is negligible.

Based on this overview, it is possible to understand the causes and the metabolic consequences of a deficiency or an excess in systemic tryptophan. Lack of tryptophan may be due to an unsuitable diet or a transport defect. In Hartnup disorder, SLC6A19 deficiency induces a decrease in intestinal absorption and renal reabsorption of tryptophan, leading to an elimination of tryptophan and derivatives in feces and urine. Tryptophan deficiency manifests by a lack of nicotinamide whose synthesis is mainly initiated by TDO in the kynurenine pathway. In normal individuals, overeating tryptophan has little impact on its blood concentration, as the excess is metabolized by TDO in the liver. Conversely, TDO deficiency causes an hypertryptophanemia whose severity depends on dietary tryptophan. In line with this observation, a defect of hepatic tryptophan transport in SLC16A10-deficient subjects also leads to hypertryptophanemia. High blood tryptophan levels in TDO-deficient subjects have neurologic and immunologic consequences. First, an excess of tryptophan induces an increase in serotonin synthesis by TPH1/2, which reduces anxiety and boosts neurogenesis. And secondly, hypertryptophanemia can compromise the peripheral tryptophan depletion triggered by IDO1 and abolish local immunosuppression.

## 8 Substrate-controlled degradation of a catabolic enzyme: A paradigm for homeostasis of other amino acids ?

In contrast with the main amino-acid sensitive signaling pathways such as GCN2 and mTOR, which unselectively sense amino-acid availability, homeostasis regulation of each amino acid seems to occur through selective metabolic or catabolic enzymes allowing to regulate their concentrations independently of each other. Enzyme activity is often directly regulated by amino-acid levels themselves. This system provides the advantage of directly stopping the synthesis of excess amino acids or the degradation of those in default, faster than via regulation of transcription or translation of the enzymes. The feedback loop regulating TDO stability is a variation along the same theme.

Common mechanisms adapting enzymatic activity to amino-acid abundance are allosteric regulations, in which the binding of a ligand is influenced by the binding of another ligand to a distant or allosteric site of the protein. These mechanisms can be separated into two categories. On the one hand, the concentration of a given amino acid can directly influence its metabolism by binding the enzymes responsible for its synthesis or degradation. This concept is particularly well illustrated by phenylalanine hydroxylase (PAH), the hepatic enzyme catalyzing the hydroxylation of L-phenylalanine to L-tyrosine (Jaffe et al., 2013). PAH is a homotetrameric protein containing six phenylalanine-binding sites: four catalytic sites, each located on a different subunit, and two exosites or allosteric activators formed at the dimer interface. Although the allosteric mechanism by which phenylalanine enhances PAH activity is not fully described, the most convincing model is that high phenylalanine levels lock the enzyme under a highly active tetrameric conformation (Jaffe et al., 2013). This positive cooperativity is believed to regulate phenylalanine homeostasis in the blood. On the other hand, the activity of an enzyme that catalyzes the degradation of a specific amino acid may be influenced by the allosteric binding of another amino acid evolving in a similar way. The best known example concerns the activity of glutamate dehydrogenase (GDH) (Li et al., 2014). Although this enzyme catalyzes the deamination of L-glutamate into alpha-ketoglutarate, its activity is positively regulated by leucine binding. During proteolysis, the increased availability of leucine, which is particularly frequent in animal proteins, would act as a signal increasing the degradation of excess glutamate. Regulation of TDO activity by tryptophan fits with an allosteric regulation. Indeed, the binding of tryptophan in exosites stabilizes the tetrameric conformation of TDO, which otherwise dissociates into inactive monomers and dimers. However, this model is distinct from classical allosteric regulation, as it involves modulation of the assembly of protein subunits. Moreover, the release of tryptophan from exosites unmasks a degron that triggers TDO degradation by the ubiquitin-proteasome system, which irrevocably interrupts tryptophan catabolism. Thus, the regulation of TDO activity involves a continuous protein turnover, which restrains more strictly tryptophan catabolism than a classical allosteric regulation.

Although many enzymes play crucial roles in amino-acid homeostasis, the tryptophan-sensing mechanism ubiquitinating TDO is the first of its kind to be described. This new mechanism could also regulate similar enzymes involved in the degradation of ingested amino acids. In a curiously similar way, Stipanuk and colleagues observed that the level of cysteine dioxygenase (CDO), the hepatic enzyme catalyzing the degradation of L-cysteine in cysteinesulfinate, decreased in mouse hepatocytes incubated in low-cysteine medium (Stipanuk et al., 2004). This decline was prevented when cysteine was supplemented in the medium. Increase in CDO level and activity worked independently of transcription, as the mRNA level of CDO remained constant. Suspecting a post-translational degradation of the protein by the ubiquitin proteasome system, the authors showed that CDO was polyubiquitinated in the absence, but not in the presence of cysteine. Accordingly, proteasome inhibition was shown to prevent CDO degradation in low cysteine conditions. These results were confirmed with human hepatocarcinoma HEPG2 cells transfected with CDO (Stipanuk et al., 2004) and *in vivo* with living rats (Dominy et al., 2006). Altogether, these observations suggest that a high concentration of cysteine in the portal vein increases the CDO level in hepatocytes, preventing hypercysteinemia. Concordant results showed that CDO-KO mice have a plasmatic cysteine concentration 2-fold higher than wild-type mice (Ueki et al., 2011). Interestingly, the same group showed that cysteamine stabilized the CDO protein level without being a substrate or an inhibitor of the enzyme (Stipanuk et al., 2004). They did not describe the molecular mechanism responsible for cysteine dioxygenase turnover, but based on our results with TDO, we would tend to assume that CDO stabilization also involves an exosite. This could be determined by crystallizing CDO with cysteamine. Such a discovery could open a framework to manipulate cysteine blood levels. Moreover, it would be interesting to define, as we did for TDO, which ubiquitin ligase(s) address(es) CDO to the proteasome.

## 9 Importance of tryptophan homeostasis

Regarding the key role of TDO in tryptophan metabolism and homeostasis, it would be expected that TDO-deficient subjects exhibit metabolic disorders. As for many other proteins, TDO-deficient humans and mice do not suffer from serious health issues, probably due to compensation mechanisms and a privileged life environment, protective and rich in food. As tryptophan degradation by TDO in the liver is the main source of nicotinamide (Figure 4), it would be expected that TDO inactivation leads to pellagra symptoms. However, Terakata and co-workers observed that TDO-KO mice sustained optimal growth and exhibited a normal weight, even when fed with a vitamin B3-free diet (Terakata et al., 2013). Subsequent analysis revealed that nicotinamide blood levels were reduced by 50% in TDO-KO mice fed without vitamin B3, but were not reduced in wild-type mice. Although TDO contributes to nicotinamide synthesis, TDO-KO mice were able to synthesize the minimum of necessary nicotinamide from the tryptophan ingested. Interestingly, TDO-deficiency induced an increase of kynurenine urinary excretion, meaning that tryptophan was more metabolized by IDO1 in extra-hepatic tissues in TDO-KO mice than in wild-type mice. By importing blood kynurenine, the liver could therefore bypass the absence of TDO. Consistent with this hypothesis, knock-out mice for quinolinate phosphoribosyl transferase, which metabolizes the tryptophan catabolites from IDO1 and TDO (Figure 4), exhibited a nicotinamide deficiency under vitamin B3 restriction (Terakata et al., 2012). It would be interesting to confirm that IDO1 compensates TDO deficiency for nicotinamide synthesis by examining double IDO1-TDO-KO mice fed without vitamin B3.

TDO-deficient mice and humans present marked hypertryptophanemia due to the non-degradation of ingested tryptophan (Kanai et al., 2009; Ferreira et al., 2017). This induces an increase of tryptophan metabolism by TPH1 and TPH2 producing 5-hydroxytryptophan, resulting in a higher concentration of serotonin in blood and brain. In mice, accumulation of the neurotransmitter was associated with neurologic and behavioral perturbations, consisting in more neurogenesis and less anxiety. Apart from these alterations, hypertryptophanemia does not seem to affect the health of TDO-deficient subjects. Therefore, we could wonder why blood tryptophanemia is so strictly contained and why it involves an expensive regulatory mechanism requiring TDO continuous turnover. The reasons remain obscure and an explanation probably resides in the less challenging environment that we and laboratory mice daily face. As explained above, tryptophan biosynthesis is energetically and enzymatically expensive, so that tryptophan is a key component in the struggle for life. Many studies reported that tryptophan depletion represses the growth of pathogenic microorganisms in mice and humans (Byrne et al., 1986b; Pfefferkorn et al., 1986a; Schmidt et al., 2009; Ganesan and Roy 2019). Therefore, maintaining a low systemic concentration of tryptophan, thanks to TDO alone or in combination with IDO1, could limit or prevent infectious diseases. Such a mechanism also exists with iron, another essential nutrient for human and pathogenic microbes, whose systemic concentration and availability are carefully controlled (Cassat and Skaar, 2013). To our knowledge, the incidence of infectious diseases has never been investigated in subjects suffering from hypertryptophanemia. Of course, the impact of pathogens is likely limited on TDO-deficient mice living in a germ-free environment or on humans living in good sanitary conditions. On the other hand, low tryptophan levels could be required so that IDO1 can locally deplete tryptophan concentration to a level sufficient to impair T-cell responses, as illustrated by the study of IDO-expressing tumors growing in TDO-KO mice (Schramme et al., 2020). Additional investigations are required to understand more precisely why blood tryptophanemia is so strictly contained.

## 10 TDO, a key target for immunotherapy

Modulating tryptophan homeostasis could be of medical interest in both directions: inhibiting tryptophan degradation to promote tumor immune rejection or increasing tryptophan catabolism to promote immune tolerance and prevent autoimmune diseases. Comprehension of the mechanism regulating TDO activity provides the opportunity to manipulate systemic tryptophanemia in both ways: using TDO inhibitors to increase it, or TDO exosite stabilizers, such as alpha-methyl-tryptophan, to reduce it. Increasing the systemic concentration of tryptophan by inhibiting hepatic TDO could overcome the tryptophan depletion inducing immunotolerance in peripheral tissues. This could be useful to promote tumor immune rejection. In support of this strategy, our group showed that TDO-KO mice treated with immune checkpoint inhibitors rejected more efficiently immunosuppressive MC38 tumors expressing IDO1 than wild-type mice (Schramme et al., 2020). Tumor rejection was associated to hypertryptophanemia, as normalization of blood tryptophanemia in TDO-KO mice fed with a low tryptophan diet abolished tumor rejection. Thus, pharmacological inhibitors of TDO could be used to overcome peripheral immune tolerance. Such inhibition should not induce severe side effects, as TDO-deficient subjects exhibit only minor phenotypic alterations.

TDO stabilizers such as alpha-methyl-tryptophan provide a new opportunity to decrease circulating tryptophan, which could promote immune tolerance and improve autoimmune diseases. This concept has never been investigated, as it was difficult to specifically induce IDO1 or TDO to promote tryptophan degradation. Our results showed that treating mice with alpha-methyl-tryptophan stably reduces the systemic level of tryptophan from 60 to 20 µM (Klaessens et al., 2021). Such a concentration is still higher than the level of 1 µM causing T-cell inhibition. As a consequence, the tryptophan depletion induced by TDO stabilization should not be sufficient to induce immune tolerance alone. Therefore, it would be interesting to investigate the tolerogenic effect of a low blood tryptophan level in an autoimmune mouse model involving IDO1, such as graft-versus-host disease (GVHD) or multiple sclerosis (Kwidzinski et al., 2005; Jasperson et al., 2008). Interestingly, blood tryptophan depletion due to permanent TDO stabilization should not induce the same symptoms as tryptophan deficiency characterized by pellagra. Indeed, blood tryptophan availability will decrease, but the synthesis of nicotinamide and NAD/P(H) from tryptophan by the TDO pathway will be maintained. Therefore, decreasing blood tryptophanemia could induce immune tolerance, without the major side effects caused by tryptophan deficiency.

## 11 Conclusion

Recent observations help to understand which mechanisms regulate homeostasis of systemic tryptophan, whose concentration is strictly maintained at 60 µM. Tryptophan catabolism by hepatic TDO plays a central role in degrading more than 90% of dietary tryptophan. Indeed, deficiency in TDO or in hepatic tryptophan transporter SLC16A10 leads to severe hypetryptophanemia. Tryptophan itself regulates TDO stability, promoting degradation of excess tryptophan or repressing degradation below physiological levels. This is achieved through the binding of tryptophan in non-catalytic exo-sites that stabilize the active tetrameric conformation of TDO. Lack of tryptophan binding in exo-sites destabilizes TDO in inactive monomers and dimers, and unmask a degron in exo-sites that promotes TDO degradation by the ubiquitin-proteasome system. Understanding these mechanisms allows to manipulate tryptophanemia in both directions, by stabilizing or inhibiting TDO. This could be of interest to modulate immune responses. This new mechanism of amino-acid homeostasis, based on substrate-regulated degradation of a catabolic enzyme, could also apply to other amino acids.
